# The Protein Network Surrounding the Human Telomere Repeat Binding Factors TRF1, TRF2, and POT1

**DOI:** 10.1371/journal.pone.0012407

**Published:** 2010-08-25

**Authors:** Richard J. Giannone, Hayes W. McDonald, Gregory B. Hurst, Rong-Fong Shen, Yisong Wang, Yie Liu

**Affiliations:** 1 Biosciences Division, Oak Ridge National Laboratory, Oak Ridge, Tennessee, United States of America; 2 Chemical Sciences Division, Oak Ridge National Laboratory, Oak Ridge, Tennessee, United States of America; 3 Vanderbilt-Ingram Cancer Center, Vanderbilt University, Nashville, Tennessee, United States of America; 4 Laboratory of Molecular Gerontology, National Institute on Aging, National Institutes of Health, Baltimore, Maryland, United States of America; 5 Medical Oncology Branch, National Cancer Institute, National Institutes of Health, Bethesda, Maryland, United States of America; Duke University, United States of America

## Abstract

Telomere integrity (including telomere length and capping) is critical in overall genomic stability. Telomere repeat binding factors and their associated proteins play vital roles in telomere length regulation and end protection. In this study, we explore the protein network surrounding telomere repeat binding factors, TRF1, TRF2, and POT1 using dual-tag affinity purification in combination with multidimensional protein identification technology liquid chromatography - tandem mass spectrometry (MudPIT LC-MS/MS). After control subtraction and data filtering, we found that TRF2 and POT1 co-purified all six members of the telomere protein complex, while TRF1 identified five of six components at frequencies that lend evidence towards the currently accepted telomere architecture. Many of the known TRF1 or TRF2 interacting proteins were also identified. Moreover, putative associating partners identified for each of the three core components fell into functional categories such as DNA damage repair, ubiquitination, chromosome cohesion, chromatin modification/remodeling, DNA replication, cell cycle and transcription regulation, nucleotide metabolism, RNA processing, and nuclear transport. These putative protein-protein associations may participate in different biological processes at telomeres or, intriguingly, outside telomeres.

## Introduction

The terminal ends of most linear eukaryotic chromosomes contain proteinaceous-DNA structures known as telomeres [1]. Telomeres are composed of double-stranded tandem repeat sequences, followed by a single-stranded, short 3′-overhang which is predicted to invade the telomeric double-stranded DNA, forming a protective cap-like structure. Disruption of this “t-loop” configuration and subsequent exposure of the 3′-overhang represent an uncapped state of telomeres [2]. Uncapped telomeres result in cell cycle arrest, cellular senescence or apoptosis and are often erroneously repaired in the form of chromosome fusions via the non-homologous end joining pathway [3], [4]. This leads to fusion-breakage-fusion cycles and chromosomal fragmentation. Therefore, the integrity of the telomere, especially in regards to its role in the protection of chromosomal attrition, is a vital component of overall genomic stability.

In mammals, telomeres are bound by shelterin, a six subunit complex composed of the telomere repeat binding factors TRF1, TRF2, POT1 and their associated proteins RAP1, TPP1, and TIN2 [5]–[7]. TRF1 and TRF2 bind to duplex telomeric DNA and anchor the shelterin along the telomere repeats [8]–[10]; POT1 binds to the single strand DNA overhang and associates with the shelterin complex [11]–[13]. TIN2 serves as the hub of the complex linking TRF1 and TRF2 [5], [14], [15] while also recruiting POT1 to the complex via TPP1 [12], [16], [17]. RAP1 associates with the telomere protein complex through its association with TRF2 [18], [19]. Telomere protein complexes and protein components are also found in other organisms, demonstrating the importance of these telomere specific proteins to telomere function [20], [21].

The telomere protein complex controls telomere length. It has been suggested that TRF1 regulates telomere length through a counting mechanism and that the interaction of POT1/TPP1 with TRF1 allows communication between the double-stranded telomeres and telomerase at the 3′-overhang [22]–[29]. The telomere repeat binding factors may also regulate telomere length by ensuring efficient telomere replication [30]–[33]. Telomere protein complex is essential in telomere capping, specifically the formation and/or regulation of the telomeric t-loop structure [2]. Telomeres that are severely or completely stripped of protective telomere repeat binding factors, such as TRF2 and POT1, evoke a DNA damage response and/or become the target of recombination repair [23], [34]–[39].

Increasing evidence suggests that telomere integrity is dependent on the ability to maintain telomere length and shield the region from recognition as damaged DNA [3], [4], [29]. These two tasks are mediated through the association of shelterin with other proteins or protein complexes. Although key components of the telomere protein complex have been identified, an in-depth picture of the associating protein networks surrounding these components has yet to be further described. A number of proteins are identified to associate with the telomere repeat binding factors, i.e. DNA repair/damage checkpoint proteins including ATM, ATR, MRE11/NBS1/RAD50 complex, components of homologous recombination or non-homologous end joining (BRCA1, KU, DNA-pkc), nucleotide excision repair/base excision repair (ERCC1/XPF, PARP1, PARP2, FEN1), DNA helicases and nucleases (WRN, BLM, Apollo, EXOL1, MUS81), and other nuclear proteins (Tankyrase 1 and 2, PIN1, PINX1, DNA topoisomerase IIIalpha, the F-box protein FBX4, nucleolar protein nucleostemin, origin replication protein ORC1, and end-binding protein EB1) ([40]–[44] and reviewed in [3], [4], [7]). Many of these proteins are actively involved in telomere length regulation, telomere DNA replication, telomere capping, and formation and/or resolution of t-loop and aberrant telomere structure.

Another aspect to consider is that these telomere-associated proteins or protein-protein associations may participate in different biological processes at telomeres. It is possible that different sets of proteins may associate with TRF1, TRF2, and POT and contribute to either telomere length regulation or telomere capping. TRF1 and/or TRF2 also regulates telomere transcription, telomere silencing, telomere sister cohesion, and the recruitment of telomere DNA to the macromolecular complexes in telomerase deficient human cells [45]–[48]. Some evidence has suggested an alternate but complementary function of telomere repeat binding factors in other cellular functions such as general DSB repair [49]–[53], neuronal gene silencing [54], and microtubule polymerization [55]. Therefore, a more complete picture of telomere-associated networks may help uncover the role of telomere repeat binding factors telomere capping/length regulation as well as cellular function outside telomeres. To address this, a novel dual-tag affinity purification system was applied to human telomere repeat binding factors TRF1, TRF2, and POT1 to garner the protein networks that associate with these proteins. Expectedly, all 6 components of shelterin were identified. Also identified were candidate proteins that are involved in a variety of cellular functions, e.g. DNA damage repair, ubiquitination, chromosome cohesion, DNA replication, and transcription regulation.

## Methods

### Gateway cloning of telomere repeat binding factors TRF1, TRF2, and POT1 into dual-tag affinity purification vectors

Human TRF2 coding sequence was cloned into an N-terminal His-tev-Strep dual-tag affinity purification vector as previously described [56]. Human TRF1 coding sequence was PCR-amplified from a pET28-hTRF1 expression vector (a generous gift from Dr. David Gilley) using primers specific for the N- and C-terminal region of the TRF1 coding sequence, flanked by either attB1 or attB2 sequences (Gateway-compatible, Invitrogen, Carlsbad. CA) and made compatible with the incorporation of an N-terminal affinity tag (via deletion of the native start codon). The primers used were as follows: (forward primer) 5′ - G GGG ACA AGT TTG TAC AAA AAA GCA GGC TTC GCG GAG GAT GTT TCC TCA G - 3′ and (reverse primer) 5′ - GGG GAC CAC TTT GTA CAA GAA AGC TGG GTC CTA GTC TTC GCT GTC TGA GGA AAT - 3′ (underline denotes start and stop codons of the TRF1 coding sequence). The PCR product was then cloned into the Gateway pDONR221 donor vector (Invitrogen, Carlsbad, CA) through the BP recombination reaction as described by the manufacturer. TRF1 was subsequently transferred from pDONR-nt-TRF1 into a Gateway-compatible destination vector, N-His-tev-Strep [56], by an LR recombination reaction according to the manufacturer's instruction. Human POT1 coding sequence was obtained from Invitrogen's Ultimate ORF Clones collection in Gateway-compatible entry vector pENTR221-hPOT1. The POT1 sequence from the entry vector was then transferred into the N-ProA-tev-Strep dual-tag affinity purification vector [56] via an LR recombination reaction as described above.

### Cell culture and stable cell line

For all experiments, human embryonic kidney 293T cells (ATCC, Manassas, VA) stably expressing both a tetracycline repressor protein (referred as 293T-REx cells) and a tetracycline-regulatable, dual-tagged fusion protein were utilized [56]. The establishment of these cell lines, each containing one of the dual-tagged telomere repeat binding factors, TRF1, TRF2, or POT1, has been previously described [56]. Briefly, 293T-REx cells were co-transfected with a dual-tag construct and pBabe-puro (a generous gift from Dr. Gerard Evans). The positive clones were identified by selection with 3 µg/ml puromycin, 800 µg/ml G418 and 5 µg/ml Blasticidin-S. The most tetracycline responsive clones for each dual-tag fusion proteins were selected and used for further experiments. All the stable cell lines were cultured at 37°C in Dulbecco's Minimum Essential Medium (DMEM; Mediatech, Inc., Manassas, VA) supplemented with 10% fetal bovine serum (FBS), penicillin/streptomycin, and L-glutamine.

### Indirect immunofluorescence in combination with telomere fluorescence in situ hybridization (TEL-FISH)

TEL-FISH was used for assessing expression and proper localization of the fusion proteins to the telomere according to previously published protocols [57] with some modifications. Briefly cells were fixed in cold methanol (Sigma) at −20°C for 10 minutes, permeabilized with 0.5% NP-40, and blocked in 1% bovine serum albumin (BSA) (IgG-free) (Sigma). Cells were immunostained with an anti-Strep antibody (Qiagen) overnight at 4°C followed by Alexa 488- labeled secondary antibody (1∶500; Molecular Probes) for one hour at 37°C and then fixed in 2% paraformaldehyde at room temperature for 10 minutes. Cells were washed with PBS for 15 minutes and then hybridized to a Cy-3-labeled (CCCTAA)_3_ PNA probe (Applied Biosystems). Fluorescent images were acquired on a Zeiss Axiophot fluorescence microscope.

### Dual-tag affinity purification of telomere repeat binding factors and associated partners

The protein purification procedures were conducted as previously described [56]. In brief, for every biological replicate, 293T-REx cells stably expressing one of the dual-tagged telomere repeat binding factors were seeded on four 15 cm culture dishes, grown to 70% confluence, and induced with 5 µg/ml of tetracycline to express the fusion protein. Cells were harvested and proteins were extracted by a modified freeze/thaw lysis procedure that aims to keep the lysate as concentrated as possible [56]. Once obtained, the crude lysate was precleared by centrifugation and the bait proteins and their associating partners were dual-tag affinity purified. Lysate for each bait protein was loaded atop beads specific to the outer affinity tag of each dual-tagged telomere repeat binding factor, i.e. Ni-NTA (Qiagen, Valencia, CA) for His-tag purification of N-HtS-TRF1 and N-HtS-TRF2; IgG Sepharose™ 6 Fast Flow (GE Healthcare, Piscataway, NJ) for ProA-tag purification of N-PtS-POT1. The bead-bound bait protein and associated proteins were washed, and then eluted from the beads with AcTEV protease (Invitrogen, Carlsbad, CA). The supernatant for each bait protein was then placed atop Strep-Tactin beads (IBA, Göttingen, Germany) and batch purified a second time. Doubly purified bait proteins and associated partners were then washed one final time, eluted with desthiobiotin, precipitated with trichloroacetic acid, and prepared for mass spectrometry analysis as described below.

### MS sample preparation and MudPIT 2D-LC-MS/MS analysis

For MS analysis, samples were prepared as originally described with modifications [58], [59]. Briefly, the trichloroacetic acid precipitated proteins were resuspended in and denatured by 8M Urea. Disulfide bonds were reduced with Tris (2-carboxyethyl) phosphine (TCEP; Bond-Breaker by Pierce, Rockford, IL) and the -SH of cysteines were alkylated with iodoacetamide (+57 Da mass shift) to prevent reformation of disulfide bonds. The reduced and denatured proteins were then digested to peptides with endoproteinase Lys-C followed by overnight trypsin digestion. The resultant peptides were acidified with formic acid, loaded onto the back column of a 3-phase MudPIT setup (reverse phase C18 for desalting, strong cation exchange for separation by positive charge, filter union, then reverse phase C18 resolving column) using a pressure cell as previously described [58], [59]. As detailed in [56], five LC-MS/MS cycles were performed per biological replicate with each consisting of a short salt pulse followed by a two-hour acetonitrile gradient to separate peptides for eventual identification via tandem MS. The chromatography was performed using an UltiMate™ LC pump (LC Packings) online with an LTQ mass spectrometer (Thermo Finnigan) outfitted with a nanospray source and operating in data dependent mode.

### MS data analysis

Tandem mass spectra were analyzed by DBDigger [60] using the International Protein Index's (IPI) human FASTA protein sequence database, version 3.25. The search algorithm was instructed to apply a static +57 Da modification to cysteine residues to compensate for the action of iodoacetamide used to alkylate free SH groups during the digestion process. The relevant search parameters were as follows: enzyme specificity was fully tryptic, a precursor mass error of 3.0 Da, a fragment mass error of 0.5 m/z, and a peptide mass range spanning 400 to 5100 Da. After data extraction from the MS/MS spectra, DTASelect was used to filter and organize the search results [60]. Peptide identification was contingent upon the following criteria: XCorr filter levels were required to be ≥23, ≥28, or ≥43, for singly-, doubly-, and triply-charged ions, respectively. DeltCN was required to be ≥0.08 and a minimum of 2 peptides was required for each protein identified. Additionally, only proteins that were absent from the control dataset were considered. The control dataset was generated by performing several pull downs in cells that expressed the vector alone to identify proteins that non-specifically bind to the affinity beads used in the purification.

### Scripting and database construction

To better handle the generated datasets, a Perl scripts were written to extract the data from the DTASelect output file (DTASelect-filter.txt) for import into a MySQL database. The parameters extracted included: gene ID, IPI number, peptide count, spectral count, sequence coverage, subcellular localization and description of the protein. The resultant Excel file allowed for a better analysis of identifications.

### Protein network visualization via Osprey

The Osprey network visualization software [61] was utilized to visualize the protein network surrounding the telomere repeat binding factors. For each bait protein, TRF1, TRF2, and POT1, the MS-identified putative associating partners were added as nodes and color-coded based on the frequency of identification (i.e. TIN2, 7 out of 8, green) for quick reference to the prevalence of the identification. Once added, Osprey was instructed to analyze previous association data within the imported nodes. To view the bait-enriched protein complexes/networks, the identified proteins for each bait protein were organized by their major function.

## Results

**Figure 1:**
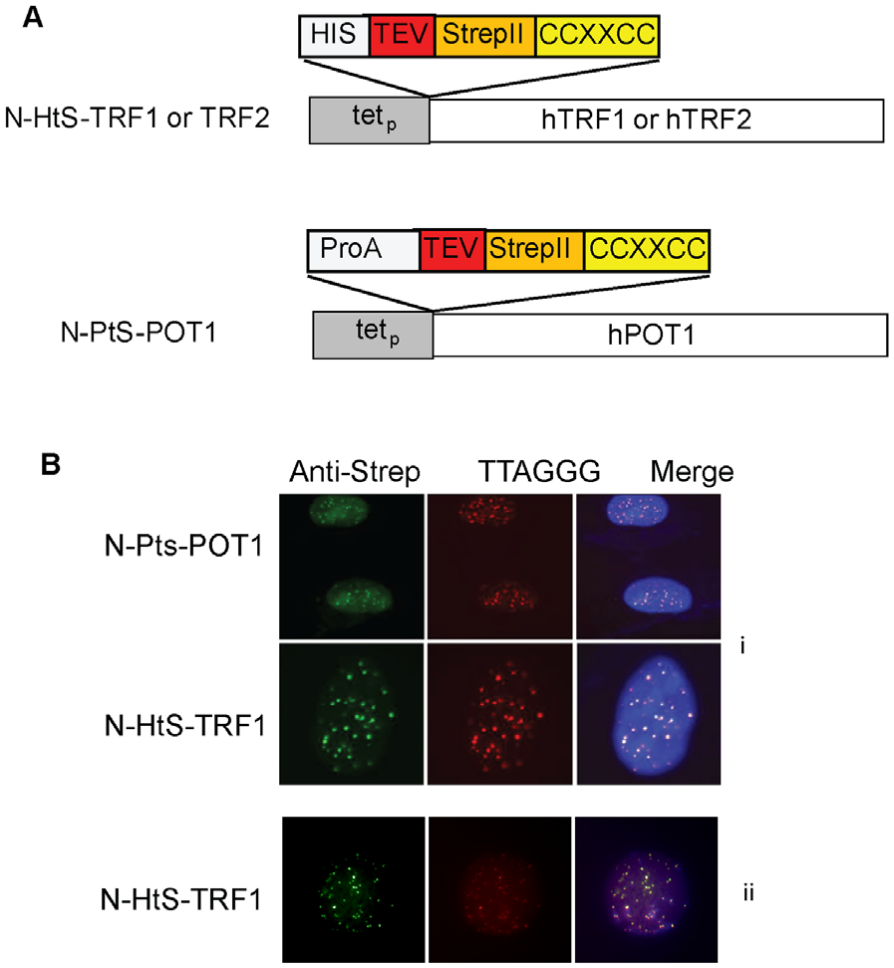
Telomeric localization of POT1 and TRF1 fusion proteins. (A) N-HtS-TRF1, N-HtS-TRF1, and N-PtS-POT1 vectors. Dual-tags and their relative positions in TRF1, TRF2, or POT1 fusions are as indicated. CCXXCC, a tetracysteine motif; S, Strep-Tactin binding peptides (StrepII-tag); t, tobacco etch virus (TEV) protease cleavage site; P, immunoglobulin G (IgG) binding domain of protein A from Staphylococcus aureus (ProA tag); HIS, 6 X histidine. (B) U2OS cells transiently (i) or 293T cells stably (ii) expressing N-HtS-TRF1 or N-PtS-POT1 were fixed and probed using a combined indirect immunostaining/telomere-FISH approach that labels Strep-tagged proteins (left panel) and the telomere (middle panel) respectively. The Merge panel indicates the degree of co-localization at near 100%.

In this study, the dual-tag affinity purification system was applied to all the telomere repeat binding factors, TRF1, TRF2, and POT1. The three bait proteins utilized in this study were N-HtS-TRF1, N-HtS-TRF2, and N-PtS-POT1 (note: The N-HtS-POT1 fusion protein failed to express in transiently transfected 293T cells). Before stable lines for each fusion protein were created, their expression and proper localization to the telomere were verified. As shown, transiently transfected HtS-TRF2, HtS-TRF1, and PtS-POT1 were successfully expressed and localized to the telomere ([56] and Figure 1). This verification was followed by stable clone creation using the above mentioned bait constructs and 293T-REx cells. Stable clones that exhibited sufficient modulation by tetracycline addition and expressed the bait protein at the endogenous protein level were selected for the analysis as previously described [56].

The bait proteins and their known associating partners after two separate purification events were eluted, precipitated, digested to peptides, and analyzed by Mud-PIT LC-MS/MS. The protein purification efficiency and quality were shown as previously [56]. For each dual-tagged bait protein, at least five biological replicates were analyzed by mass spectrometry, except for TRF2, which had eight total replicates. However, three of these replicates had an exposure to sham- and ionizing radiation prior to cell lysis. The tandem mass spectra for each sample were then searched against the human IPI protein database version 3.25 with DBDigger. The identified proteins in each sample were filtered to eliminate “the contaminants” presented in 293T-REx cells expressing vector alone, and then sorted by DTASelect and listed in Table S1. Comparisons among different sample runs (or datasets) were performed, which allowed us to visualize known associating partners as well as proteins that appear to associate with these components and/or the telomere in general. Additional features i.e. cellular compartment and proposed function were included. This extra data provides an “at-a-glance” view of the identified proteins, allowing for assessments such as the frequency of identification and whether that identification is significant based on the additional information.

The number of peptides and total spectra collected for each bait protein identified in each sample are shown (Table 1). Based on spectral count, the bait protein was usually the most highly identified protein in each control-subtracted sample, evidence for the efficacy of the pulldown. With regards to the combined dataset and the number of times each protein was identified out of 18 total runs, all six shelterin components were identified (Tables 2 and S1). Five of six components were identified at a rate that ranked them within the top 10 most frequently observed proteins, while the sixth component, TRF1, was observed within the top 15 proteins. The most abundantly identified, non-bait proteins were TIN2 (13/18), RAP1 (12/18), and TPP1 (11/18), which are known shelterin components. The frequency of identification also supports the current understanding regarding the interconnectedness among the shelterin components. According to previous studies, TIN2 acts as a general hub, interacting with TRF1, TRF2, and TPP1/POT1 [5], [12], [14], [15]. Our data agrees with the sentiment that TIN2 serves as the lynchpin of the shelterin complex. TIN2 was the most abundantly identified protein, appearing in 13 of 18 datasets, co-purifying most frequently with TRF2 (7 of 8) and POT1 (4 of 5) and less frequently with TRF1 (2 of 5) (Table S1). Additionally, TPP1 and TIN2 were repeatedly found in the POT1 datasets (4 of 5) with significantly more spectra obtained compared to the other two bait proteins (Table S1); another general indicator of enrichment as the more protein there is in a sample, the more MS/MS spectra obtained. POT1 can interact directly with TRF2 which interacts directly with RAP1 [13], [18], [19]. Although frequently identified in both the TRF2 and POT1 pull downs, the identification of RAP1 in the POT1 pull down is most likely through TRF2, as evident by the abundance of spectra collected for RAP1 in the TRF2 pull down. However, one cannot exclude the possibility that RAP1 transiently interacts directly with POT1. Using HtS-TRF1 as bait, the shelterin components were identified at lower frequencies relative to the other two telomere repeat binding factors. The most significant identifications were TIN2 (2 of 5), POT1 (2 of 5), and TPP1 (2 of 5), corroborating the existence of a subcomplex consisting of TRF1/TIN2/TPP1/POT1 (Table S1). Although this data could indicate that the addition of an HtS dual-tag on TRF1's N-terminus may block its association with other members of the complex, this may not be the case as the co-purification of endogenous TRF1 via HtS-TRF2 or PtS-POT1 was equally as low.

**Table 1:** Bulk MudPIT MS/MS data for each dual-tag affinity purified telomere binding protein after searching with DBDigger and filtering with DTASelect.

Bait	Peptides	Spectral Count
***TRF1***		
HtS-TRF1-01	33	47
HtS-TRF1-02	33	48
HtS-TRF1-03	32	93
HtS-TRF1-04	80	328
HtS-TRF1-NL	33	88
***TRF2***		
HtS-TRF2-01	61	413
HtS-TRF2-02	57	262
HtS-TRF2-03	79	192
HtS-TRF2-04	52	182
HtS-TRF2-NL	66	723
***POT1***		
PtS-POT1-01	49	459
PtS-POT1-02	57	444
PtS-POT1-03	37	171
PtS-POT1-04	43	255

HtS = His-tev-Strep, PtS = ProA-tev-Strep, NL = nuclear lysate. 1–4: whole cell lysate.

**Table 2:** Spectral count data for all identified members of the shelterin complex organized by bait protein.

Bait = TRF1					Bait = TRF2								Bait = POT1					Count	Protein
1	2	3	4	NL	1	2	3	4	NL	irC	ir-1	ir-2	1	2	3	4	NL		
			10	6	5	2	5	11	2	3		5	8	20	13	20		13	TIN2
		5			**413**	**192**	**1220**	**723**	**262**	**182**	**1239**	**1244**			30	7	24	12	TRF2
					175	124	124	335	128	114	117	179		2	4	2	10	12	RAP1
			8	4	3	7		5		3	3		19	41	16	30		11	TPP1
			21	3		11		9				3	**459**	**444**	**171**	**255**	**15**	10	POT1
**47**	**48**	**93**	**328**	**88**				7									3	7	TRF1

Bolded rows indicate the bait's appearance in its own MudPIT MS/MS runs. Count represents the frequency of the identification for each member of the shelterin when the data was merged (18 total sets). irC: Untreated. ir1 and 2: exposure to 2 and 10 Grey ionizing radiation. NL: nuclear lysate. 1–4: whole cell lysate.

**Figure 2:**
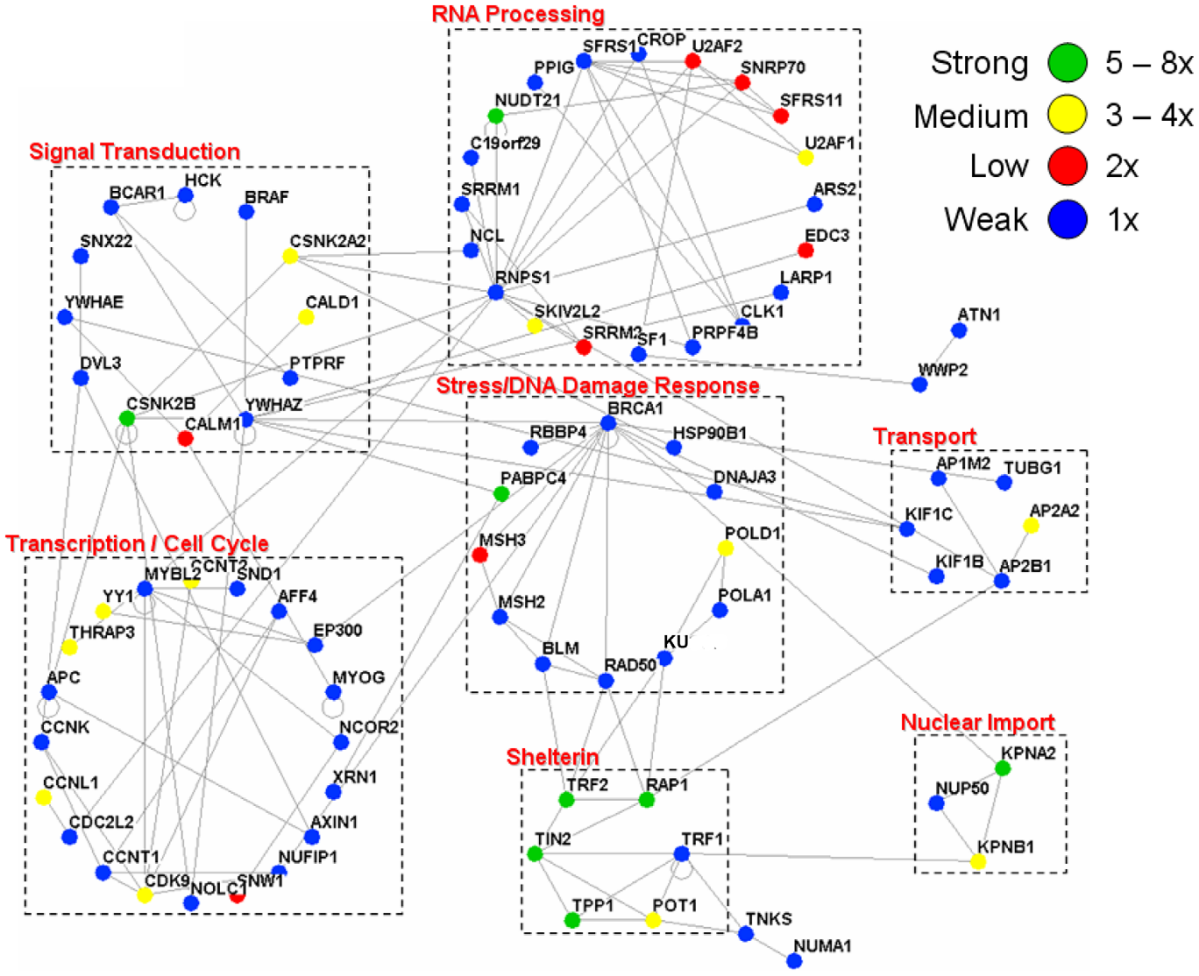
The association network surrounding human telomere repeat binding factor, TRF2. Network diagram representing all MS-identified proteins that can be connected back to TRF2. Edges were drawn between known associating proteins. The frequency of identification is listed in the key: Green = 5 to 8 times, yellow = 3 to 4 times, red = 2 times, blue = 1 time, out of a total of 8 samples. Identified proteins are grouped by function.

**Figure 3:**
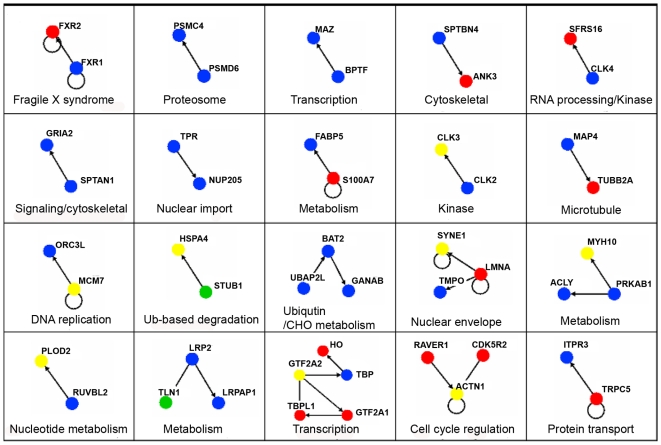
MS-identified proteins that did not connect back to TRF2, but connected to other identified proteins by this study. The same color-coded key applies as Figure 2. Proteins identified in only one of the runs were excluded in Table S1.

**Figure 4:**
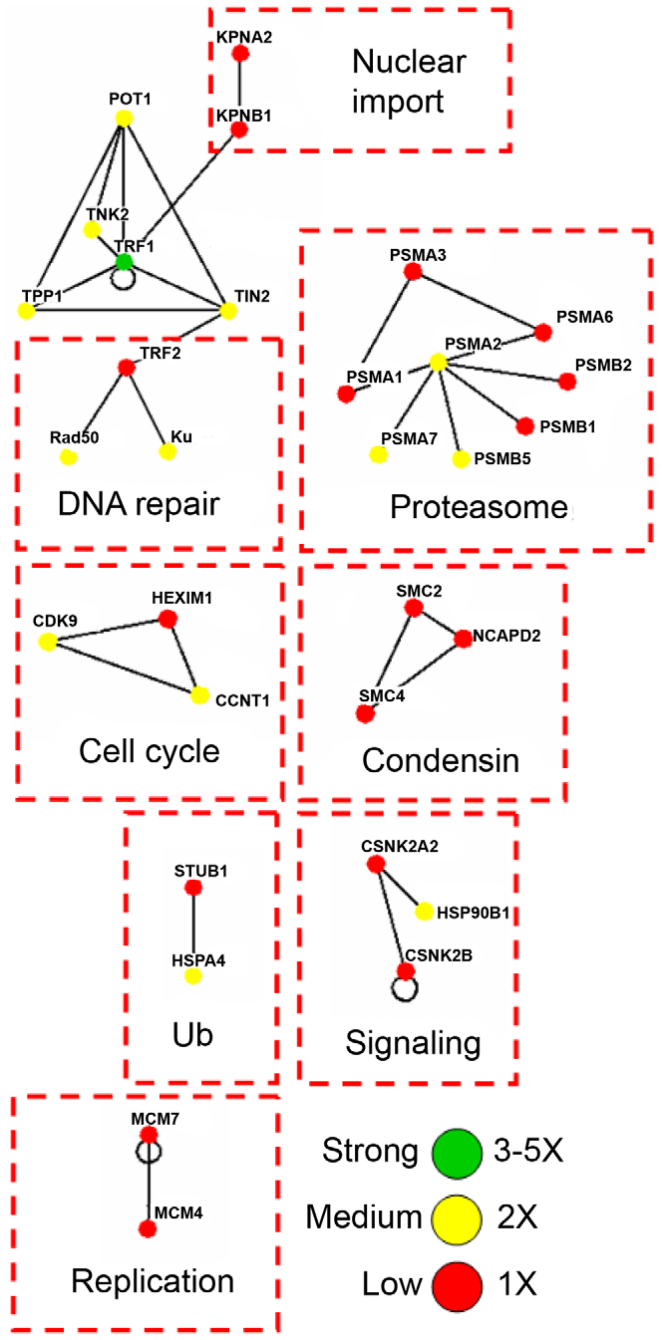
The putative associating proteins of TRF1 which can be grouped into several functional categories. Green = 3 to 5 times, Yellow = 2 times, and Red = 1 time. Proteins identified in only one of the runs were excluded in Table S1.

**Figure 5:**
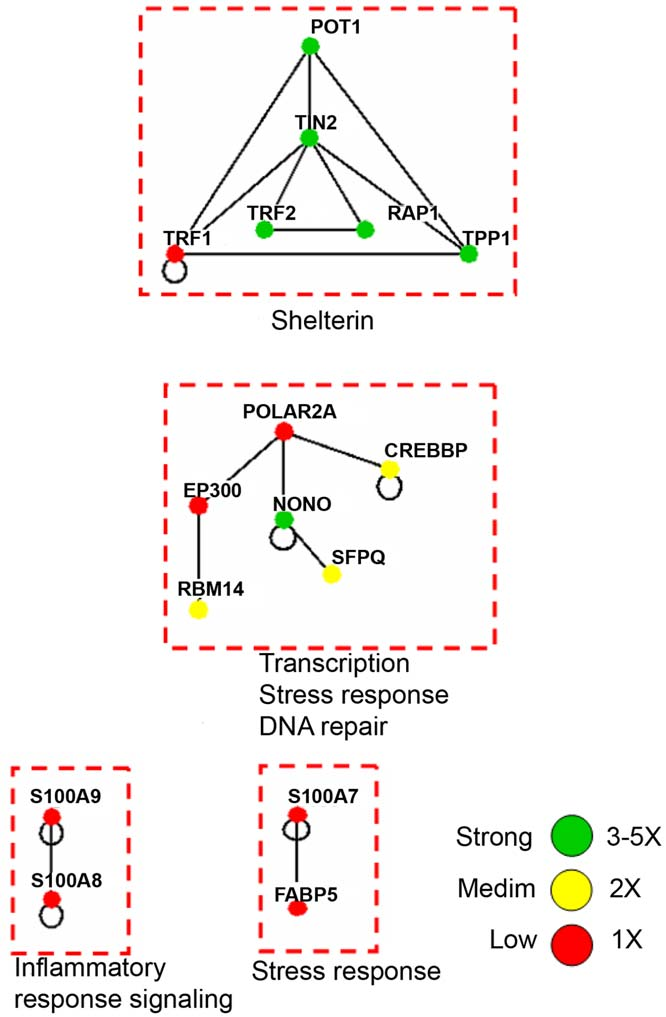
The putative associating proteins of POT1 which can be grouped into several functional categories. Green = 3 to 5 times, Yellow = 2 times, and Red = 1 time. Proteins identified in only one of the runs were excluded in Table S1.

In addition to shelterin components, known TRF1 and TRF2 associating proteins were also confidently detected, including tankyrase 1, PARP1, BLM, WRN, RAD50, DNA-pkc, Ku70/Ku86, and ATM (Table S1 and [56]). The other non-redundant proteins identified in dual-tag affinity purified TRF2 (n = 5), TRF1 (n = 5), and POT1 (n = 5) were listed as being potential associating proteins (Table S1). To better visualize the putative associating partners for each bait protein, the data for each dual-tag purification was exported to Osprey [61] and linked together based on the human BioGRID database (http://www.thebiogrid.org). Osprey is able to assemble the MS-identified proteins in terms of a protein network, linking together known associations to better visualize the degree of the association. For each specific bait protein, nodes were organized by functional grouping with color indicative of the prevalence/frequency of the association: green (high), yellow (med), red (low), and blue (very low). For the TRF2 association diagram, two subsections were illustrated: (i) a connected diagram that shows identified proteins that can be traced back to TRF2 by some degree of separation (Figure 2) and (ii) an unconnected diagram that represents identified protein networks/complexes that do not connect back to the bait protein based on previous association data (Figure 3). For each bait protein, several functional categories were represented: DNA damage response, cell cycle/transcription, RNA processing, signal transduction, transport, nuclear import, ubiquitin-based protein degradation, and DNA replication/nucleotide metabolism for TRF2 (Figures 2 and 3); nuclear import machinery, DNA replication, ubiquitin-based protein degradation, signaling, cell cycle regulation, and chromosome condensation for TRF1 (Figure 4); and transcription, the cellular response to stress, and DNA repair for POT1 (Figure 5).

It should be understood that identified proteins may be cell-type specific and do not necessarily interact directly. In fact, it is highly conceivable that many co-purifying proteins are secondary, tertiary, or even quaternary associations. The capture of these non-directly associating proteins most likely depends on both the affinity of the association(s) as well as the enhanced stability imparted by multiple associations. Therefore, the identification of associating proteins should be expanded to include associating complexes as well. These network diagrams are indicators of (i) the function of a bait protein, represented by co-purifying protein functional groups, (ii) novel associating complexes/networks with indication as to which component may be the direct link, and (iii) purification frequency (color as an indicator) and interconnectedness, lending evidence towards whether a proposed association is direct or indirect. Although these frequencies are helpful, they should be used with caution. For example, intuitively, we may regard a “green” identification as being a positive, strong associating partner, while “blue” identification is weak and/or possibly a false-positive due to lack of representation. However, a protein identified at a very low frequency could be indicative of a transient association. Conversely, a protein identified at a high frequency could be indicative of a non-specific protein that co-purifies with the bait protein all the time, though attempts were made to minimize these spurious hits (e.g. control-dataset subtraction).

Because TRF2 is critical in telomere capping and associates with many DNA repair/damage checkpoint proteins, we explored its association in a 293T-REx clone stably expressing HtS-TRF2 construct. Cells were exposed to different dosage of gamma radiation (2Gy and 10 Gy). Compared with sham-irradiated cells (irC), a number of proteins uniquely copurified with TRF2 in the treated cells (Table S2). Some common proteins were detected after 2Gy and 10Gy irradiation treatment, but unique proteins were also found in each treatment, possibly due to distinct cellular response to 2Gy and 10Gy irradiation. It remains to be evaluated if TRF2 may associate with different sets of protein partners under these specific damage conditions.

## Discussion

In this study we explored the protein network surrounding the human telomere repeat binding factors TRF1, TRF2, and POT1. To accomplish this task, we utilized a previously developed dual-tag affinity purification system to purify each telomere repeat binding factor along with their associated partners. Bait and associating protein analysis was conducted via MudPIT LC-MS/MS and bioinformatics to identify the most relevant associations over several biological replicates.

As expected, the bait proteins were identified in each replicate and usually occupied the top position when proteins were ranked by spectral count. Per bait protein, different numbers of non-redundant proteins were identified for TRF2, TRF1, and POT1, most of which consisted of known nuclear proteins. We could not rule out the possibility that the baits may also associate with cytoplasmic proteins. For example, it was recently discovered that the association of POT1, TIN2, and TPP1 can be observed in the cytoplasm [62]. In addition, novel associations which would have otherwise been missed can be identified using this broader dataset. For instance, one of the proteins identified in the TRF2 pull down analysis was KPNA2/importin alpha 1. It is hypothesized that this protein only associates with its cargo in the cytoplasm, but not in the nucleus [63]. A previous study has shown that the nuclear import of TRF1 is inhibited by importin alpha [64]. Similarly, further inquiry into this association suggests that KPNA2 acts as a negative regulator of TRF2 nuclear localization (Giannone et al, unpublished data). Thus, choosing to analyze only the nuclear lysate, although producing generally improved bait and known associating partner capture, would miss some otherwise interesting associating partners.

The protein network analysis for each bait protein yielded interesting results. For TRF2, many potential associating partners fell into several functional classifications. The most prevalent categories included the shelterin complex, DNA damage response and repair, transcription/cell cycle regulation, signal transduction, RNA processing, protein transport, nuclear import, ubiquitin-based degradation, and DNA replication. Also, several other categories were represented that do not appear in the network connection diagrams including chromosome cohesion and chromatin modification/remodeling. Several of the above mentioned functional groups have been previously identified to relate in some way to TRF2's function. For example, TRF2 is associated with a number of DNA damage-sensing and repair proteins ([51], [65], [66] and reviewed in [3], [4], [7]). In fact, there were a total of 22 proteins identified that relate, in some way, to the DNA damage response or to DNA repair including but not limited to: Rad50 (known [66], HR), BRCA1 (known [40]), tumor suppressor/genome surveillance), Ku86 (known [67], NHEJ), MSH2 and MSH3 (mismatch repair), and DDB1 (nucleotide excision repair). Other functional categories, including DNA replication (11 proteins identified), ubiquitin-based protein degradation (30 proteins), sumoylation, chromosome cohesion (6 proteins), and chromatin remodeling/modification (21 proteins) have been previously linked to TRF1, TRF2 or the telomere [30], [46], [47], [54], [68]-[71].

In comparison to TRF2, the other telomere repeat binding factors did not co-purify as many potentially novel associating proteins, though they each identified all or most of the components of the shelterin complex. Interestingly, the connectivity of TRF1 and POT1 co-purifying proteins was substantially less than TRF2. This perhaps indicates that these proteins participate in fewer cellular pathways than TRF2. Nevertheless, the major TRF1 co-purifying complexes fell into the following function categories: the shelterin, nuclear import machinery, DNA replication, ubiquitin-based protein degradation, signaling, cell cycle regulation, and chromosome condensation. Some functional groups have been reported to relate to TRF1's function. It has been shown that TRF1 is imported into the nucleus via classical nuclear import, regulated by both KPNB1 and KPNA2 [64]. Additionally, recent reports have implicated TRF1 in DNA damage-mediated cell cycle arrest [72] and ubiquitin-based protein degradation [68], [73]. Moreover, members of the structural maintenance of chromosomes (SMC) family have also been linked to the telomere and TRF1 [47]. TRF1 and TIN2 each bind to a cohesion ortholog, suggesting an association between cohesions and telomeric chromatin in human cells [46]. Finally, TRF1's involvement in DNA replication has been suggested [32], [33], [69].

In regards to POT1, the identification of potential associating complexes and proteins were even less than what was identified for TRF1, suggesting either the loss of associating proteins with lower affinities, interference from non-specific proteins (i.e. the leaching of immunoglobin components from the beads specific to ProA purification), or perhaps the notion that this protein may play less of a role in processes outside the telomere. Whatever the case, POT1 co-purified several subcomplexes including the shelterin, those involved in transcription, the cellular response to stress, and DNA repair.

It is noteworthy that some known shelterin-interacting proteins are not found in this study, such as TRF2/Apollo and TRF1/FBX4. Several reasons may contribute to the discrepancy. It has been shown that the expression of endogenous Apollo and FBX4 is not detectable or low in 293T or human tissues [74], [75]. It is therefore likely that these proteins as well as other shelterin-interacting proteins that are expressed at low levels may not be detectable by our method. In addition, we could not rule out the possibilities that these proteins may associate shelterin components transiently or with low affinity and these protein-protein interactions may be cell-type specific.

In this study, we have probed the association network surrounding each telomere repeat binding factor TRF1, TRF2, and POT1. The complete identification of all known shelterin components as well as identification of known interacting partners for each bait protein lends validity to our approach. Interestingly, the shelterin co-purification data presented here confirms previous assessments regarding the overall architecture of complex as well as cellular function at or outside the telomere. The association analysis provided in this study has identified leads for future analysis.

## Supporting Information

Table S1 Combined list of co-purifying proteins identified, organized by the bait proteins.
(0.10 MB PDF)

Table S2 Combined list of TRF2-copurifying proteins identified in untreated and radiation treated cells.
(0.09 MB PDF)
