# Supplementary material for: The Protein Network Surrounding the Human Telomere Repeat Binding Factors TRF1, TRF2, and POT1
Source: PLoS One. 2010 Aug 25;5(8):e12407. doi: 10.1371/journal.pone.0012407 (PMC2928292; doi:10.1371/journal.pone.0012407)
Supplement: Table S2 — Combined list of TRF2-copurifying proteins identified in untreated and radiation treated cells. (0.09 MB PDF) [file pone.0012407.s002.pdf]

Table S2: Combined list of TRF2-copurifying proteins indentified in untreated and radiation treated cells

TRF2-1	TRF2-2	TRF2-3	TRF2-4	TRF2-NL	TRF2-irC	TRF2-ir1	TRF2-ir2	Gene	Count	Total sequence coverage of the protein	UniprotID	Cellular Location	Description	Function	Keywords
4(5)*	2(2)	2(2)	3(3)	10(11)	4(5)		4(5)	**TIN2**	13	74.8	Q9BSI4	Nucleus	shelterin component	Involved in the regulation of telomere length. Prevents excessive telomere elongation.	Alternative splicing; Chromosomal protein; Nucleus; Telomere.
61(413)	57(192)	79(262)	52(182)	66(723)	89(1220)	86(1239)	98(1244)	**TRF2**	12	86.8	Q15554	Nucleus	shelterin component	Binds the telomeric double-stranded TTAGGG repeat. Protects against end-to-end fusion of chromosomes and plays a role in successful progression through the cell division cycle.	3D-structure; Alternative splicing; Cell cycle; Chromosomal protein; DNA-binding; Nucleus; Phosphoprotein; Telomere.
36(175)	34(124)	47(128)	39(114)	60(335)	37(124)	31(117)	39(179)	**RAP1**	12	78.7	Q9NYB0	Nucleus	shelterin component	May play a role in telomere length regulation.	3D-structure; Chromosomal protein; Nucleus; Phosphoprotein; Telomere.
3(3)	3(7)		3(3)	4(5)		3(3)		**TPP1**	11	44.7	Q96AP0	Nucleus	shelterin component	Plays a role of telomeric regulator as component of TRF1 complex controlling telomere length; controls POT1 telomeric recruitement and telomere elongation by inhibition of telomerase activity. May play a role in organogenesis.	3D-structure; Alternative splicing; Chromosomal protein; DNA-binding; Nucleus; Telomere.
3(3)	8(18)	15(24)	14(31)		4(5)	6(9)	3(5)	**ZC3HAV1L**	10	57	Q96H79		Zinc finger CCCH-type, antiviral 1-like protein.		
	5(5)	14(15)	10(11)	4(4)	3(4)	4(5)	3(4)	**RBM15B**	10	32.9	Q8NDT2	-	Putative RNA-binding protein 15B (RNA-binding motif protein 15B).		Nucleus; Phosphoprotein; Repeat; RNA-binding.
	3(11)			8(9)			3(3)	**POT1**	10	75.2	Q9NUX5	Nucleus	Protection of telomeres protein 1 (hPot1) (POT1-like telomere end- binding protein).	Component of the telomerase ribonucleoprotein (RNP) complex that is essential for the replication of chromosome termini. Is a component of the double-stranded telomeric DNA- binding TRF1 complex which is involved in the regulation of telomere length by cis-inhibition of telomerase. Also acts as a single-stranded telomeric DNA-binding protein and thus may act as a downstream effector of the TRF1 complex and may transduce information about telomere maintenance and/or length to the telomere terminus. Binds to two or more telomeric single-stranded 5'-TTAGGG-3' repeats (G-strand) and with high specificity to a minimal telomeric single-stranded 5'-TAGGGTTAG-3' sequence. Binds telomeric single-stranded sequences internally or at proximity of a 3'-end. Its activity is TERT dependent but it does not increase TERT activity.	3D-structure; Alternative splicing; Chromosomal protein; DNA-binding; Nucleus; Polymorphism; Telomere.
	2(2)	2(2)	2(2)		2(3)	2(3)	5(6)	**PLAT**	9	16.5	P00750	Extracellular	Tissue-type plasminogen activator precursor (EC 3.4.21.68) (tPA) (t- PA) (t-plasminogen activator) (Alteplase) (Reteplase) [Contains: Tissue-type plasminogen activator chain A; Tissue-type plasminogen activator chain B].	Converts the abundant, but inactive, zymogen plasminogen to plasmin by hydrolyzing a single Arg-Val bond in plasminogen. By controlling plasmin-mediated proteolysis, it plays an important role in tissue remodeling and degradation, in cell migration and many other physiopathological events. Play a direct role in facilitating neuronal migration.	3D-structure; Alternative splicing; Cleavage on pair of basic residues; Direct protein sequencing; EGF-like domain; Glycoprotein; Hydrolase; Kringle; Pharmaceutical; Plasminogen activation; Polymorphism; Protease; Repeat; Secreted; Serine protease; Signal; Zymogen.
2(2)	6(7)	9(10)	6(9)		3(3)	4(5)	3(3)	**DKFZp761H0716**	8	44.1	Q8N3N9		Pyrroline-5-carboxylate reductase (EC 1.5.1.2) (Fragment).		Amino-acid biosynthesis; NADP; Oxidoreductase; Proline biosynthesis.

2(2)	2(2)	3(3)	2(2)		5(6)	3(4)	4(4)	**KPNA2**	8	23.6	P52292	Cytoplasm; Nucleus; Plasma membrane; Nucleolus	Importin subunit alpha-2 (Karyopherin subunit alpha-2) (SRP1-alpha) (RAG cohort protein 1).	Functions in nuclear protein import as an adapter protein for nuclear receptor KPNB1. Binds specifically and directly to substrates containing either a simple or bipartite NLS motif. Docking of the importin/substrate complex to the nuclear pore complex (NPC) is mediated by KPNB1 through binding to nucleoporin FxFG repeats and the complex is subsequently translocated through the pore by an energy requiring, Ran- dependent mechanism. At the nucleoplasmic side of the NPC, Ran binds to importin-beta and the three components separate and importin-alpha and -beta are re-exported from the nucleus to the cytoplasm where GTP hydrolysis releases Ran from importin. The directionality of nuclear import is thought to be conferred by an asymmetric distribution of the GTP- and GDP-bound forms of Ran between the cytoplasm and nucleus.	3D-structure; Cytoplasm; Host-virus interaction; Nucleus; Phosphoprotein; Polymorphism; Protein transport; Repeat; Transport.
				6(7)				**TRF1**	7	71.5	P54274	Nucleus; Cytoplasm	Telomeric repeat-binding factor 1 (TTAGGG repeat-binding factor 1) (NIMA-interacting protein 2) (Telomeric protein Pin2/TRF1).	Binds the telomeric double-stranded TTAGGG repeat and negatively regulates telomere length. Involved in the regulation of the mitotic spindle.	3D-structure; ADP-ribosylation; Alternative splicing; Cell cycle; Cell division; Chromosomal protein; Direct protein sequencing; DNA-binding; Mitosis; Nucleus; Phosphoprotein; Telomere.
2(3)	4(5)	15(31)	8(12)			2(2)	2(2)	**STUB1**	7	16.5	Q9UNE7	Cytoplasm; Endoplasmic reticulum	STIP1 homology and U box-containing protein 1 (EC 6.3.2.-) (STIP1 homology and U-box-containing protein 1) (Carboxy terminus of Hsp70- interacting protein) (E3 ubiquitin-protein ligase CHIP) (CLL- associated antigen KW-8) (Antigen NY-CO-7).	Modulates the activity of several chaperone complexes, including Hsp70, Hsc70 and Hsp90. Has E3 ubiquitin-protein ligase activity and targets misfolded chaperone substrates towards proteasomal degradation. Mediates transfer of non-canonical short ubiquitin chains to HSPA8 that have no effect on HSPA8 degradation.	Alternative splicing; Cytoplasm; Ligase; Phosphoprotein; Repeat; TPR repeat; Ubl conjugation; Ubl conjugation pathway.
2(3)	3(3)		5(7)	2(2)				**YY1**	7	27.1	P25490	Nucleus	Transcriptional repressor protein YY1 (Yin and yang 1) (YY-1) (Delta transcription factor) (NF-E1).	Multifunctional transcription factor that exhibits positive and negative control on a large number of cellular and viral genes by binding to sites overlapping the transcription start site. May play an important role in development and differentiation. The function of YY1 as an activator or a repressor is specified by the presence of other proteins. For example it acts as a repressor in absence of adenovirus E1A protein but as an activator in its presence.	3D-structure; Activator; Direct protein sequencing; DNA-binding; Metal-binding; Nucleus; Phosphoprotein; Repeat; Repressor; Transcription; Transcription regulation; Zinc; Zinc-finger.
	2(6)	8(10)	9(13)			3(3)		**PRR8**	7	36	Q9NSV0	-	Proline-rich protein 8.		Coiled coil; Phosphoprotein; Polymorphism.
	2(2)	4(5)	4(5)		2(2)	2(4)	3(5)	**PABPC4**	7	20.6	Q13310	Cytoplasm	Polyadenylate-binding protein 4 (Poly(A)-binding protein 4) (PABP 4) (Inducible poly(A)-binding protein) (iPABP) (Activated-platelet protein 1) (APP-1).	Binds the poly(A) tail of mRNA. May be involved in cytoplasmic regulatory processes of mRNA metabolism. Can probably bind to cytoplasmic RNA sequences other than poly(A) in vivo (By similarity).	Alternative splicing; Cytoplasm; Methylation; Phosphoprotein; Repeat; RNA-binding.

23(52)	10(14)	17(29)	15(20)		6(8)	2(2)	4(4)	**MCCC2**	7	50.3	Q9HCC0	Mitochondrion	Methylcrotonoyl-CoA carboxylase beta chain, mitochondrial precursor (EC 6.4.1.4) (3-methylcrotonyl-CoA carboxylase 2) (MCCase subunit beta) (3-methylcrotonyl-CoA:carbon dioxide ligase subunit beta) (3- methylcrotonyl-CoA carboxylase non-biotin-containing subunit).		Alternative splicing; Disease mutation; Ligase; Mitochondrion; Transit peptide.
	4(6)	5(5)	5(8)		2(2)	2(2)		**CSNK2B**	6	32.8	P67870	Nucleus; Cytoplasm	Casein kinase II subunit beta (CK II beta) (Phosvitin) (G5a).	Participates in Wnt signaling (By similarity). Plays a complex role in regulating the basal catalytic activity of the alpha subunit.	3D-structure; Phosphoprotein; Wnt signaling pathway.
2(2)	10(13)	4(5)	9(14)			2(2)		**BMP2K**	6	19.6	Q9NSY1	-	BMP-2-inducible protein kinase (EC 2.7.11.1) (BIKe).	May be involved in osteoblast differentiation.	Alternative splicing; ATP-binding; Kinase; Nucleotide-binding; Nucleus; Phosphoprotein; Serine/threonine-protein kinase; Transferase.
9(14)	7(8)	13(13)	10(12)				2(2)	**CYFIP1**	6	18.8	Q7L576	Cytoplasm; Nucleus	Cytoplasmic FMR1-interacting protein 1 (Specifically Rac1-associated protein 1) (Sra-1) (p140sra-1).	Involved in formation of membrane ruffles and lamellipodia protrusions and in axon outgrowth. Binds to F-actin but not to RNA.	Actin-binding; Alternative splicing; Cell junction; Cell projection; Cell shape; Cytoplasm; Developmental protein; Differentiation; Direct protein sequencing; Neurogenesis; Synapse; Synaptosome.
	7(10)		5(7)	4(4)	2(2)			**DDX3X**	6	23.3	O00571	Nucleus; Cytoplasm; Nucleolus	ATP-dependent RNA helicase DDX3X (EC 3.6.1.-) (DEAD box protein 3, X- chromosomal) (Helicase-like protein 2) (HLP2) (DEAD box, X isoform).	ATP-dependent RNA helicase. Acts as a cofactor for XPO1- mediated nuclear export of incompletely spliced HIV-1 Rev RNAs. Also involved in HIV-1 replication. Interacts specifically with hepatitis C virus core protein resulting in a change in intracellular location.	Acetylation; ATP-binding; Cytoplasm; Direct protein sequencing; DNA-binding; Helicase; Host-virus interaction; Hydrolase; Nucleotide-binding; Nucleus; Phosphoprotein; Polymorphism; RNA-binding.
	3(5)	4(5)	3(6)		3(3)	2(3)	2(2)	**SF3B4**	6	23.1	Q15427	Nucleus	Splicing factor 3B subunit 4 (Spliceosome-associated protein 49) (SAP 49) (SF3b50) (Pre-mRNA-splicing factor SF3b 49 kDa subunit).	Subunit of the splicing factor SF3B required for 'A' complex assembly formed by the stable binding of U2 snRNP to the branchpoint sequence (BPS) in pre-mRNA. Sequence independent binding of SF3A/SF3B complex upstream of the branch site is essential, it may anchor U2 snRNP to the pre-mRNA. May also be involved in the assembly of the 'E' complex. SF3B4 has been found in complex 'B' and 'C' as well. Belongs also to the minor U12- dependent spliceosome, which is involved in the splicing of rare class of nuclear pre-mRNA intron.	3D-structure; mRNA processing; mRNA splicing; Nucleus; Phosphoprotein; Repeat; RNA-binding; Spliceosome.
		2(2)	2(2)					**S100A8**	5	32.3	P05109	Cytoplasm; Extracellular; Plasma membrane	Protein S100-A8 (S100 calcium-binding protein A8) (Calgranulin-A) (Migration inhibitory factor-related protein 8) (MRP-8) (Cystic fibrosis antigen) (CFAG) (P8) (Leukocyte L1 complex light chain) (Calprotectin L1L subunit) (Urinary stone protein band A).	Expressed by macrophages in chronic inflammations. Also expressed in epithelial cells constitutively or induced during dermatoses. May interact with components of the intermediate filaments in monocytes and epithelial cells.	3D-structure; Calcium; Direct protein sequencing; Repeat.
	2(2)		2(2)				3(3)	**HSPA4**	5	16.7	P34932	Golgi apparatus; Plasma membrane; Extracellular	Heat shock 70 kDa protein 4 (Heat shock 70-related protein APG-2) (HSP70RY).		ATP-binding; Cytoplasm; Direct protein sequencing; Nucleotide-binding; Phosphoprotein; Stress response.
	4(5)		9(10)				2(2)	**ACTN1**	5	17.3	P12814	Cytoplasm	Alpha-actinin-1 (Alpha-actinin cytoskeletal isoform) (Non-muscle alpha-actinin-1) (F-actin cross-linking protein).	F-actin cross-linking protein which is thought to anchor actin to a variety of intracellular structures. This is a bundling protein.	3D-structure; Actin-binding; Calcium; Cytoplasm; Cytoskeleton; Direct protein sequencing; Phosphoprotein; Repeat.

3(3)	5(5)	3(3)	7(7)					**MCM7**	5	23.8	P33993	Nucleus; Nucleolus	DNA replication licensing factor MCM7 (CDC47 homolog) (P1.1-MCM3).	Acts as a factor that allows the DNA to undergo a single round of replication per cell cycle. Required for DNA replication and cell proliferation. Required for S-phase checkpoint activation upon UV-induced damage.	Alternative splicing; ATP-binding; Cell cycle; DNA replication; DNA-binding; Nucleotide-binding; Nucleus; Phosphoprotein; Polymorphism; Transcription; Transcription regulation.
2(2)	3(4)	2(2)	4(6)					**DDB1**	5	9.3	Q16531	Nucleus; Cytoplasm; Nucleolus	DNA damage-binding protein 1 (Damage-specific DNA-binding protein 1) (UV-damaged DNA-binding factor) (DDB p127 subunit) (DNA damage-binding protein a) (DDBa) (UV-damaged DNA-binding protein 1) (UV-DDB 1) (Xeroderma pigmentosum group E-complementing protein) (XPCe) (XPE- binding factor) (XPE-BF) (HBV X-associated protein 1) (XAP-1).	Plays a role in DNA repair by forming with DDB2 the UV-damaged DNA-binding protein complex (UV-DDB). Binds to pyrimidine dimers. Component of the RBX1-CUL4-DDB2 ubiquitin ligase. Required for histone H3 and histone H4 ubiquitination in response to ultraviolet and may be important for subsequent DNA repair.	3D-structure; Cytoplasm; DNA damage; DNA repair; DNA-binding; Host-virus interaction; Nucleus; Phosphoprotein; Polymorphism; Ubl conjugation pathway.
3(5)	12(30)	3(3)	11(30)					**PRDX4**	5	16.7	A6NG45	Cytoplasm; Endoplasmic reticulum; Golgi apparatus; Nucleus	Uncharacterized protein PRDX4.		
	2(11)		1(11)					**RASGEF1C**	5	6.9	Q8N431	-	Ras-GEF domain-containing family member 1C.	Guanine nucleotide exchange factor (GEF) (By similarity).	Alternative splicing; Guanine-nucleotide releasing factor.
	2(2)	2(2)	5(6)					**CDK9**	5	28.2	P50750	Nucleus; Cytoplasm; Nucleolus	Cell division protein kinase 9 (EC 2.7.11.22) (EC 2.7.11.23) (Cyclin- dependent kinase 9) (Serine/threonine-protein kinase PITALRE) (C-2K) (Cell division cycle 2-like protein kinase 4).	Member of the cyclin-dependent kinase pair (CDK9/cyclin- T) complex, also called positive transcription elongation factor b (P-TEFb), which facilitates the transition from abortive to production elongation by phosphorylating the CTD (C-terminal domain) of the large subunit of RNA polymerase II (RNAP II), SUPT5H and RDBP. The CDK9/cyclin-K complex has also a kinase activity toward CTD of RNAP II and can substitute for P-TEFb in vitro.	3D-structure; Alternative splicing; ATP-binding; Kinase; Nucleotide-binding; Nucleus; Phosphoprotein; Polymorphism; Serine/threonine-protein kinase; Transcription; Transcription regulation; Transferase.
	4(6)	7(10)	5(6)					**FBXO21**	5	19.7	Q8IUQ5	-	FBXO21 protein (F-box protein 21, isoform CRA_b).		
	5(10)	7(12)	9(17)		2(3)			**BAIAP2L1**	5	39.3	Q9UHR4	-	Brain-specific angiogenesis inhibitor 1-associated protein 2-like protein 1 (BAI1-associated protein 2-like protein 1).	May function as adapter protein (Potential).	Coiled coil; Phosphoprotein; Polymorphism; SH3 domain.
	2(2)	4(4)	9(9)					**CHD8**	5	13.4	Q9HCK8	-	Chromodomain-helicase-DNA-binding protein 8 (EC 3.6.1.-) (ATP- dependent helicase CHD8) (CHD-8) (Helicase with SNF2 domain 1).	Probable transcription regulator.	3D-structure; Alternative splicing; ATP-binding; Chromatin regulator; DNA-binding; Helicase; Hydrolase; Nucleotide-binding; Nucleus; Phosphoprotein; Repeat; Transcription; Transcription regulation.
			7(8)	4(6)				**TOX4**	5	15.9	O94842		TOX high mobility group box family member 4 (Epidermal Langerhans cell protein LCP1).		DNA-binding; Nucleus; Phosphoprotein.
	3(6)	5(9)	5(7)			2(2)		**CLK3**	5	8.4	P49761	Nucleus	Dual specificity protein kinase CLK3 (EC 2.7.12.1) (CDC-like kinase 3).	Phosphorylates serine- and arginine-rich (SR) proteins of the spliceosomal complex. May be a constituent of a network of regulatory mechanisms that enable SR proteins to control RNA splicing. Phosphorylates serines, threonines and tyrosines.	3D-structure; Alternative splicing; ATP-binding; Cytoplasm; Kinase; Nucleotide-binding; Nucleus; Phosphoprotein; Serine/threonine-protein kinase; Transferase; Tyrosine-protein kinase.

	8(8)	5(5)	8(9)				2(2)	**SEC16A**	5	12.8	O15027		SEC16 homolog A.		Alternative splicing; Phosphoprotein; Polymorphism.
2(2)		2(2)	2(2)					**DNAJB11**	5	13.4	Q9UBS4	Endoplasmic reticulum	DnaJ homolog subfamily B member 11 precursor (ER-associated dnaJ protein 3) (ErJ3) (ER-associated Hsp40 co-chaperone) (hDj9) (PWP1-interacting protein 4).		Chaperone; Endoplasmic reticulum; Phosphoprotein; Polymorphism; Signal.
2(2)	4(5)	5(5)	4(6)				2(2)	**TLN1**	5	7.6	Q9Y490	Extracellular; Plasma membrane; Cytoplasm	Talin-1.	Probably involved in connections of major cytoskeletal structures to the plasma membrane. High molecular weight cytoskeletal protein concentrated at regions of cell-substratum contact and, in lymphocytes, at cell-cell contacts (By similarity).	3D-structure; Cell projection; Cytoplasm; Cytoskeleton; Direct protein sequencing; Membrane; Phosphoprotein; Polymorphism; Structural protein.
3(9)		3(3)			4(7)	3(6)	4(7)	**MYO10**	5	2.5	Q9HD67	Plasma membrane; Cytoplasm	Myosin-X (Unconventional myosin-10).	Myosins are actin-based motor molecules with ATPase activity. Unconventional myosins serve in intracellular movements. Their highly divergent tails are presumed to bind to membranous compartments, which would be moved relative to actin filaments (By similarity). Plays a role in regions of dynamic actin.	Actin-binding; ATP-binding; Coiled coil; Motor protein; Myosin; Nucleotide-binding; Phosphoprotein; Repeat.
	2(9)					1(10)		**ABCD1**	5	1.7	P33897	Peroxisome	ATP-binding cassette sub-family D member 1 (Adrenoleukodystrophy protein) (ALDP).	Probable transporter. The nucleotide-binding fold acts as an ATP-binding subunit with ATPase activity.	ATP-binding; Disease mutation; Glycoprotein; Membrane; Nucleotide-binding; Peroxisome; Transmembrane; Transport.
			2(2)					**derp12**	4	34.1	Q8TE01		DERP12 (Dermal papilla derived protein 12).		FAD; Flavoprotein; NADP; Oxidoreductase; Redox-active center.
	2(2)				2(2)			**S100A7**	4	45	P31151	Cytoplasm; Endoplasmic reticulum; Nucleus; Plasma membrane; Extracellular	Protein S100-A7 (S100 calcium-binding protein A7) (Psoriasin).		3D-structure; Acetylation; Calcium; Cytoplasm; Direct protein sequencing; Metal-binding; Repeat; Secreted; Zinc.
	2(12)		2(11)					**HSP90Bb**	4	16.1	Q58FF8		Heat shock protein 90Bb.		ATP-binding; Chaperone; Nucleotide-binding; Stress response.

	2(2)					3(3)	5(6)	**KPNB1**	4	11.8	Q14974	Cytoplasm; Nucleus; Extracellular	Importin subunit beta-1 (Karyopherin subunit beta-1) (Nuclear factor P97) (Importin 90).	Functions in nuclear protein import, either in association with an adapter protein, like an importin-alpha subunit, which binds to nuclear localization signals (NLS) in cargo substrates, or by acting as autonomous nuclear transport receptor. Acting autonomously, serves itself as NLS receptor. Docking of the importin/substrate complex to the nuclear pore complex (NPC) is mediated by KPNB1 through binding to nucleoporin FxFG repeats and the complex is subsequently translocated through the pore by an energy requiring, Ran-dependent mechanism. At the nucleoplasmic side of the NPC, Ran binds to importin-beta and the three components separate and importin-alpha and -beta are re- exported from the nucleus to the cytoplasm where GTP hydrolysis releases Ran from importin. The directionality of nuclear import is thought to be conferred by an asymmetric distribution of the GTP- and GDP-bound forms of Ran between the cytoplasm and nucleus. Mediates autonomously the nuclear import of ribosomal proteins RPL23A, RPS7 and RPL5. Binds to a beta-like import receptor binding (BIB) domain of RPL23A. In association with IPO7 mediates the nuclear import of H1 histone. In vitro, mediates nuclear import of H2A, H2B, H3 and H4 histones. In case of HIV-1 infection, binds and mediates the nuclear import of HIV-1 Rev. Imports PRKCI into the nucleus.	3D-structure; Acetylation; Cytoplasm; Direct protein sequencing; Host-virus interaction; Nucleus; Protein transport; Repeat; Transport; Ubl conjugation.
		7(8)	9(11)				2(2)	**USP9X**	4	8.3	Q93008	Cytoplasm	Probable ubiquitin carboxyl-terminal hydrolase FAF-X (EC 3.1.2.15) (Ubiquitin thioesterase FAF-X) (Ubiquitin-specific-processing protease FAF-X) (Deubiquitinating enzyme FAF-X) (Fat facets protein-related, X-linked) (Ubiquitin-specific protease 9, X chromosome).	May function as a ubiquitin-protein or polyubiquitin hydrolase involved both in the processing of ubiquitin precursors and of ubiquitinated proteins. May therefore play an important role regulatory role at the level of protein turnover by preventing degradation of proteins through the removal of conjugated ubiquitin.	Alternative splicing; Hydrolase; Phosphoprotein; Protease; Thiol protease; Ubl conjugation pathway.
								**RPS2**	4	33.8	P15880	Ribosome; Nucleolus	40S ribosomal protein S2 (S4) (LLRep3 protein).		
	4(4)	6(7)	2(2)					**CSNK2A2**	4	39.1	P19784	Nucleus; Cytoplasm; Plasma membrane; Nucleolus	Casein kinase II subunit alpha' (EC 2.7.11.1) (CK II).	Casein kinases are operationally defined by their preferential utilization of acidic proteins such as caseins as substrates. The alpha and alpha' chains contain the catalytic site. Participates in Wnt signaling. CK2 phosphorylates 'Ser-392' of p53/TP53 following UV irradiation.	ATP-binding; Kinase; Nucleotide-binding; Serine/threonine-protein kinase; Transferase; Wnt signaling pathway.
2(2)			3(3)	2(2)				**PLOD2**	4	27.2	O00469	Endoplasmic reticulum	Procollagen-lysine,2-oxoglutarate 5-dioxygenase 2 precursor (EC 1.14.11.4) (Lysyl hydroxylase 2) (LH2).	Forms hydroxylysine residues in -Xaa-Lys-Gly- sequences in collagens. These hydroxylysines serve as sites of attachment for carbohydrate units and are essential for the stability of the intermolecular collagen cross-links.	Alternative splicing; Dioxygenase; Disease mutation; Endoplasmic reticulum; Glycoprotein; Iron; Membrane; Metal-binding; Oxidoreductase; Signal; Vitamin C.

	2(2)	3(3)	2(2)					**RING1**	4	14.6	Q06587	Nucleus; Nucleolus	E3 ubiquitin-protein ligase RING1 (EC 6.3.2.-) (Polycomb complex protein RING1) (RING finger protein 1).	Constitutes one of the E3 ubiquitin-protein ligases that mediate monoubiquitination of 'Lys-119' of histone H2A, thereby playing a central role in histone code and gene regulation. H2A 'Lys-119' ubiquitination gives a specific tag for epigenetic transcriptional repression and participates in X chromosome inactivation of female mammals. Essential component of the Polycomb group (PcG) multiprotein PRC1 complex, a complex required to maintain the transcriptionally repressive state of many genes, including Hox genes, throughout development. PcG PRC1 complex act via chromatin remodeling and modification of histones, rendering chromatin heritably changed in its expressibility. Compared to RNF2/RING2, it does not have the main E3 ubiquitin ligase activity on histone H2A, and it may rather act as a modulator of RNF2/RING2 activity.	Alternative splicing; Chromatin regulator; Ligase; Metal-binding; Nucleus; Repressor; Transcription; Transcription regulation; Ubl conjugation pathway; Zinc; Zinc-finger.
	2(2)	2(3)	6(7)					**ZNF281**	4	13.6	Q9Y2X9	Nucleus	Zinc finger protein 281 (Zinc finger DNA-binding protein 99) (Transcription factor ZBP-99) (GC-box-binding zinc finger protein 1).	Involved in transcriptional regulation. Represses the transcription of a number of genes including gastrin and ornithine decarboxylase. Binds to the G-rich box in the enhancer region of these genes.	DNA-binding; Metal-binding; Nucleus; Phosphoprotein; Polymorphism; Repeat; Repressor; Transcription; Transcription regulation; Zinc; Zinc-finger.
	4(4)	3(3)	7(7)					**FOXJ3**	4	22.6	Q9UPW0	Nucleus	Forkhead box protein J3.		Alternative splicing; DNA-binding; Nucleus; Transcription; Transcription regulation.
	2(2)	2(2)	4(4)					**LARP7**	4	24.3	Q4G0J3		La-related protein 7 (La ribonucleoprotein domain family member 7).		Alternative splicing; Phosphoprotein; RNA-binding.
	6(6)	4(4)	10(11)					**IRS4**	4	16.9	O14654	Cytoplasm; Plasma membrane	Insulin receptor substrate 4 (IRS-4).		Receptor.
		4(4)	3(3)	3(3)				**SAP30BP**	4	22.6	Q9UHR5	-	SAP30-binding protein (Transcriptional regulator protein HCNGP).	Induces cell death. May act as a transcriptional corepressor of a gene related to cell survival. May be involved in the regulation of beta-2-microglobulin genes.	Alternative splicing; Apoptosis; Nucleus; Repressor; Transcription; Transcription regulation.
2(8)	3(3)	2(6)						**CHD5**	4	3.2	Q8TDI0	Nucleus	Chromodomain-helicase-DNA-binding protein 5 (EC 3.6.1.-) (ATP- dependent helicase CHD5) (CHD-5).	May play a role in the development of the nervous system and the pathogenesis of neural tumors.	ATP-binding; Chromatin regulator; DNA-binding; Helicase; Hydrolase; Metal-binding; Nucleotide-binding; Nucleus; Polymorphism; Repeat; Transcription; Transcription regulation; Zinc; Zinc-finger.
2(2)		2(2)		2(2)				**DST**	4	1.2	Q5TBT2	Nucleus; Plasma membrane; Extracellular	Dystonin.		Calcium.
2(8)	3(6)	3(8)	4(5)					**SNRPD1**	4	27.7	P62314	Cytoplasm; Nucleus	Small nuclear ribonucleoprotein Sm D1 (snRNP core protein D1) (Sm-D1) (Sm-D autoantigen).	May act as a charged protein scaffold to promote snRNP assembly or strengthen snRNP-snRNP interactions through nonspecific electrostatic contacts with RNA.	3D-structure; Direct protein sequencing; Methylation; mRNA processing; mRNA splicing; Nucleus; Repeat; Ribonucleoprotein; Spliceosome; Systemic lupus erythematosus.
4(4)	2(2)	2(2)	4(5)					**UGDH**	4	27.9	O60701	-	UDP-glucose 6-dehydrogenase (EC 1.1.1.22) (UDP-Glc dehydrogenase) (UDP-GlcDH) (UDPGDH).	Involved in the biosynthesis of glycosaminoglycans; hyaluronan, chondroitin sulfate, and heparan sulfate.	3D-structure; NAD; Oxidoreductase; Phosphoprotein.
2(3)	2(6)	4(6)	2(4)					**ERH**	4	31.7	P84090	Nucleus; Nucleolus	Enhancer of rudimentary homolog.	May have a role in the cell cycle.	3D-structure.

8(8)	10(11)	11(11)	8(8)					**PC**	4	27	P11498	Mitochondrion	Pyruvate carboxylase, mitochondrial precursor (EC 6.4.1.1) (Pyruvic carboxylase) (PCB).	Pyruvate carboxylase catalyzes a 2-step reaction, involving the ATP-dependent carboxylation of the covalently attached biotin in the first step and the transfer of the carboxyl group to pyruvate in the second. Catalyzes in a tissue specific manner, the initial reactions of glucose (liver, kidney) and lipid (adipose tissue, liver, brain) synthesis from pyruvate.	ATP-binding; Biotin; Disease mutation; Gluconeogenesis; Ligase; Lipid synthesis; Manganese; Mitochondrion; Multifunctional enzyme; Nucleotide-binding; Phosphoprotein; Transit peptide.
5(5)	2(3)	3(3)	2(3)					**MYO1C**	4	8.9	Q4LE56	Plasma membrane; Cytoplasm	MYO1C variant protein (Fragment).		
3(3)	5(8)	12(15)	5(7)					**PITRM1**	4	24.8	Q5JRX3	Extracellular	Presequence protease, mitochondrial precursor (EC 3.4.24.-) (hPreP) (Pitrilysin metalloproteinase 1) (Metalloprotease 1) (hMP1).	ATP-independent protease that degrades mitochondrial transit peptides after their cleavage. Also degrades other unstructured peptides. Specific for peptides in the range of 10 to 65 residues. Able to degrade amyloid beta A4 (APP) protein when it accumulates in mitochondrion, suggesting a link with Alzheimer disease. Shows a preference for cleavage after small polar residues and before basic residues, but without any positional preference.	Alternative splicing; Hydrolase; Metal-binding; Metalloprotease; Mitochondrion; Polymorphism; Protease; Transit peptide; Zinc.
6(8)	3(4)	17(24)	9(10)					**MYH10**	4	15.2	P35580	Cytoplasm	Myosin-10 (Myosin heavy chain 10) (Myosin heavy chain, non-muscle IIb) (Non-muscle myosin heavy chain IIb) (NMMHC II-b) (NMMHC-IIB) (Cellular myosin heavy chain, type B) (Non-muscle myosin heavy chain-B) (NMMHC- B).	Cellular myosin that appears to play a role in cytokinesis, cell shape, and specialized functions such as secretion and capping.	Actin-binding; Alternative splicing; ATP-binding; Calmodulin-binding; Cell shape; Coiled coil; Motor protein; Myosin; Nucleotide-binding; Phosphoprotein.
1(43)					1(15)	1(27)	1(26)	**ATP2B1**	4	1.6	P20020	Plasma membrane	Plasma membrane calcium-transporting ATPase 1 (EC 3.6.3.8) (PMCA1) (Plasma membrane calcium pump isoform 1) (Plasma membrane calcium ATPase isoform 1).	This magnesium-dependent enzyme catalyzes the hydrolysis of ATP coupled with the transport of calcium out of the cell.	Alternative splicing; ATP-binding; Calcium; Calcium transport; Calmodulin-binding; Hydrolase; Ion transport; Magnesium; Membrane; Metal-binding; Nucleotide-binding; Phosphoprotein; Polymorphism; Transmembrane; Transport.
1(43)					1(15)	1(27)	1(26)	**ATP2B3**	4	1.6	Q16720	Plasma membrane	Plasma membrane calcium-transporting ATPase 3 (EC 3.6.3.8) (PMCA3) (Plasma membrane calcium pump isoform 3) (Plasma membrane calcium ATPase isoform 3).	This magnesium-dependent enzyme catalyzes the hydrolysis of ATP coupled with the transport of calcium out of the cell.	Alternative splicing; ATP-binding; Calcium; Calcium transport; Calmodulin-binding; Hydrolase; Ion transport; Magnesium; Membrane; Metal-binding; Nucleotide-binding; Phosphoprotein; Polymorphism; Transmembrane; Transport.

	3(4)	4(4)	5(7)			2(2)		**CARM1**	4	18.3	Q86X55	Nucleus	Histone-arginine methyltransferase CARM1 (EC 2.1.1.125) (EC 2.1.1.-) (Protein arginine N-methyltransferase 4) (Coactivator-associated arginine methyltransferase 1).	Methylates (mono- and asymmetric dimethylation) the guanidino nitrogens of arginyl residues in several proteins involved in DNA packaging, transcription regulation, and mRNA stability. Recruited to promoters upon gene activation together with histone acetyltransferases from EP300/P300 and p160 families, methylates histone H3 at 'Arg-17' and activates transcription via chromatin remodeling. During nuclear hormone receptor activation and TCF7L2/TCF4 activation, acts synergically with EP300/P300 and either one of the p160 histone acetyltransferases NCOA1/SRC1, NCOA2/GRIP1 and NCOA3/ACTR or CTNNB1/beta-catenin to activate transcription. During myogenic transcriptional activation, acts together with NCOA3/ACTR as a coactivator for MEF2C. During monocyte inflammatory stimulation, acts together with EP300/P300 as a coactivator for NF-kappa-B. Also seems to be involved in p53/TP53 transcriptional activation. Methylates EP300/P300, both at 'Arg-2142', which may loosen its interaction with NCOA2/GRIP1, and at 'Arg-580' and 'Arg-604' in the KIX domain, which impairs its interaction with CREB and inhibits CREB-dependent transcriptional activation. Also methylates arginine residues in RNA-binding proteins PABPC1, ELAVL1 and ELAV4, which may affect their mRNA-stabilizing properties and the half-life of their target mRNAs.	Alternative splicing; Chromatin regulator; Cytoplasm; Host-virus interaction; Methyltransferase; Nucleus; S-adenosyl-L-methionine; Transcription; Transcription regulation; Transferase.
	4(4)		4(5)		2(2)	4(5)		**SLTM**	4	18.7	Q9NWH9	-	SAFB-like transcription modulator (Modulator of estrogen-induced transcription).	When overexpressed, acts as a general inhibitor of transcription that eventually leads to apoptosis (By similarity).	Alternative splicing; Apoptosis; Coiled coil; Nucleus; Phosphoprotein; Polymorphism; Repressor; RNA-binding; Transcription; Transcription regulation.
	2(4)		5(7)			2(2)	2(2)	**NT5DC2**	4	15.6	Q9H857		5'-nucleotidase domain-containing protein 2.		Alternative splicing; Polymorphism.
	2(2)	2(2)	4(4)					**THRAP3**	4	10.2	Q9Y2W1	Nucleus	Thyroid hormone receptor-associated protein 3 (Thyroid hormone receptor-associated protein complex 150 kDa component) (Trap150).	Plays a role in transcriptional coactivation.	Activator; ATP-binding; Direct protein sequencing; Nucleotide-binding; Nucleus; Phosphoprotein; Polymorphism; Receptor; Transcription; Transcription regulation.
			2(2)			2(2)	2(2)	**CALD1**	4	13	Q05682	Cytoplasm; Plasma membrane	Caldesmon (CDM).	Actin- and myosin-binding protein implicated in the regulation of actomyosin interactions in smooth muscle and nonmuscle cells (could act as a bridge between myosin and actin filaments). Stimulates actin binding of tropomyosin which increases the stabilization of actin filament structure. In muscle tissues, inhibits the actomyosin ATPase by binding to F-actin. This inhibition is attenuated by calcium-calmodulin and is potentiated by tropomyosin. Interacts with actin, myosin, two molecules of tropomyosin and with calmodulin. Also play an essential role during cellular mitosis and receptor capping.	Actin-binding; Alternative splicing; Calmodulin-binding; Muscle protein; Phosphoprotein; Repeat.
						2(17)		**TMPRSS13**	4	6.8	Q9BYE2	Extracellular	Transmembrane protease, serine 13 (EC 3.4.21.-) (Mosaic serine protease) (Membrane-type mosaic serine protease).		Alternative splicing; Glycoprotein; Hydrolase; Membrane; Polymorphism; Protease; Repeat; Serine protease; Signal-anchor; Transmembrane.

		2(2)						**Ku80**	3	10.8	P13010	Nucleus; Cytoplasm; Nucleolus	ATP-dependent DNA helicase 2 subunit 2 (EC 3.6.1.-) (ATP-dependent DNA helicase II 80 kDa subunit) (Lupus Ku autoantigen protein p86) (Ku86) (Ku80) (86 kDa subunit of Ku antigen) (Thyroid-lupus autoantigen) (TLAA) (CTC box-binding factor 85 kDa subunit) (CTCBF) (CTC85) (Nuclear factor IV) (DNA-repair protein XRCC5).	Single stranded DNA-dependent ATP-dependent helicase. Has a role in chromosome translocation. The DNA helicase II complex binds preferentially to fork-like ends of double-stranded DNA in a cell cycle-dependent manner. It works in the 3'-5' direction. Binding to DNA may be mediated by p70. Involved in DNA nonhomologous end joining (NHEJ) required for double-strand break repair and V(D)J recombination. The Ku p70/p86 dimer acts as regulatory subunit of the DNA-dependent protein kinase complex DNA-PK by increasing the affinity of the catalytic subunit PRKDC to DNA by 100-fold. The Ku p70/p86 dimer is probably involved in stabilizing broken DNA ends and bringing them together. The assembly of the DNA-PK complex to DNA ends is required for the NHEJ ligation step. In association with NARG1, the Ku p70/p86 dimer binds to the osteocalcin promoter and activates osteocalcin expression.	3D-structure; ATP-binding; Direct protein sequencing; DNA damage; DNA recombination; DNA repair; DNA-binding; Helicase; Hydrolase; Nucleotide-binding; Nucleus; Phosphoprotein; Polymorphism; Systemic lupus erythematosus; Ubl conjugation.
	2(2)							**HSP90B1**	3	19.6	P14625	Endoplasmic reticulum	Endoplasmin precursor (Heat shock protein 90 kDa beta member 1) (94 kDa glucose-regulated protein) (GRP94) (gp96 homolog) (Tumor rejection antigen 1).	Molecular chaperone that functions in the processing and transport of secreted proteins.	Calcium; Chaperone; Direct protein sequencing; Endoplasmic reticulum; Glycoprotein; Phosphoprotein; Signal.
			2(2)					**ACLY**	3	14.1	P53396	Cytoplasm	ATP-citrate synthase (EC 2.3.3.8) (ATP-citrate (pro-S-)-lyase) (Citrate cleavage enzyme).	ATP citrate-lyase is the primary enzyme responsible for the synthesis of cytosolic acetyl-CoA in many tissues. Has a central role in de novo lipid synthesis. In nervous tissue it may be involved in the biosynthesis of acetylcholine.	ATP-binding; Cytoplasm; Lipid synthesis; Magnesium; Metal-binding; Nucleotide-binding; Phosphoprotein; Polymorphism; Transferase.
	2(2)					2(2)		**PRPS1L1**	3	9.5	P21108	-	Ribose-phosphate pyrophosphokinase 3 (EC 2.7.6.1) (Phosphoribosyl pyrophosphate synthetase III) (PRS-III) (Phosphoribosyl pyrophosphate synthetase 1-like 1).		Direct protein sequencing; Kinase; Magnesium; Metal-binding; Nucleotide biosynthesis; Transferase.
	2(3)		4(6)					**IDH3A**	3	20.6	P50213	Mitochondrion; Extracellular	Isocitrate dehydrogenase [NAD] subunit alpha, mitochondrial precursor (EC 1.1.1.41) (Isocitric dehydrogenase) (NAD(+)-specific ICDH).		Alternative splicing; Direct protein sequencing; Magnesium; Manganese; Metal-binding; Mitochondrion; NAD; Oxidoreductase; Transit peptide; Tricarboxylic acid cycle.
			5(5)				3(3)	**HSPH1**	3	12.4	Q92598	Cytoplasm	Heat shock protein 105 kDa (Heat shock 110 kDa protein) (Antigen NY- CO-25).	Prevents the aggregation of denatured proteins in cells under severe stress, on which the ATP levels decrease markedly. Inhibits HSPA8/HSC70 ATPase and chaperone activities (By similarity).	Alternative splicing; ATP-binding; Cytoplasm; Nucleotide-binding; Phosphoprotein; Stress response.
					2(2)			**SPTAN1**	3	2.2	A6NG51	Cytoplasm; Plasma membrane	Uncharacterized protein SPTAN1.		SH3 domain.
	3(4)				2(2)			**MACROD2**	3	12	A1Z1Q3		MACRO domain-containing protein 2.		Alternative splicing.
	4(5)		5(5)					**BAG3**	3	18.6	O95817	Cytoplasm	BAG family molecular chaperone regulator 3 (Bcl-2-associated athanogene 3) (BAG-3) (Bcl-2-binding protein Bis) (Docking protein CAIR-1).	Inhibits the chaperone activity of HSP70/HSC70 by promoting substrate release. Has anti-apoptotic activity.	Apoptosis; Chaperone; Phosphoprotein; Repeat.

			2(2)					**TNKS**	3	17	O95271	Golgi apparatus; Nucleus; Cytoplasm; Endosome	Tankyrase-1 (EC 2.4.2.30) (TANK1) (Tankyrase I) (TNKS-1) (TRF1- interacting ankyrin-related ADP-ribose polymerase).	May regulate vesicle trafficking and modulate the subcellular distribution of SLC2A4/GLUT4-vesicles. Has PARP activity and can modify TERF1, and thereby contribute to the regulation of telomere length.	ADP-ribosylation; Alternative splicing; ANK repeat; Chromosomal protein; Cytoplasm; Glycosyltransferase; Golgi apparatus; Membrane; mRNA transport; NAD; Nuclear pore complex; Nucleus; Phosphoprotein; Protein transport; Repeat; Telomere; Transferase; Translocation; Transport.
			2(2)					**CCNT1**	3	8.7	O60563	Nucleus	Cyclin-T1 (CycT1) (Cyclin-T).	Regulatory subunit of the cyclin-dependent kinase pair (CDK9/cyclin-T1) complex, also called positive transcription elongation factor B (P-TEFb), which is proposed to facilitate the transition from abortive to productive elongation by phosphorylating the CTD (carboxy-terminal domain) of the large subunit of RNA polymerase II (RNA Pol II). In case of HIV or SIV infections, binds to the transactivation domain of the viral nuclear transcriptional activator, Tat, thereby increasing Tat's affinity for the transactivating response RNA element (TAR RNA). Serves as an essential cofactor for Tat, by promoting RNA Pol II activation, allowing transcription of viral genes.	Acetylation; Cell cycle; Cell division; Coiled coil; Cyclin; Direct protein sequencing; Host-virus interaction; Nucleus; Transcription; Transcription regulation.
		7(10)	2(3)					**MYH9**	3	5.9	P35579	Cytoplasm; Nucleolus	Myosin-9 (Myosin heavy chain 9) (Myosin heavy chain, non-muscle IIa) (Non-muscle myosin heavy chain IIa) (NMMHC II-a) (NMMHC-IIA) (Cellular myosin heavy chain, type A) (Non-muscle myosin heavy chain-A) (NMMHC- A).	Cellular myosin that appears to play a role in cytokinesis, cell shape, and specialized functions such as secretion and capping.	Acetylation; Actin-binding; Alport syndrome; ATP-binding; Calmodulin-binding; Cataract; Cell shape; Coiled coil; Deafness; Direct protein sequencing; Disease mutation; Motor protein; Myosin; Nucleotide-binding; Phosphoprotein; Polymorphism.
2(2)		7(11)						**TBPL1**	3	22	P62380	Nucleus; Cytoplasm	TATA box-binding protein-like protein 1 (TBP-like protein 1) (TATA box-binding protein-related factor 2) (TBP-related factor 2) (STUD protein) (21 kDa TBP-like protein).	Does not bind the TATA box. Has DNA-binding ability.	Cytoplasm; DNA-binding; Nucleus.
					2(2)			**RPS8**	3	17.9	P62241	Cytoplasm; Nucleolus	40S ribosomal protein S8.		Direct protein sequencing; Phosphoprotein; Ribonucleoprotein; Ribosomal protein.
						3(6)		**SF1**	3	10.7	Q15637	Nucleus	Splicing factor 1 (Zinc finger protein 162) (Transcription factor ZFM1) (Zinc finger gene in MEN1 locus) (Mammalian branch point-binding protein mBBP) (BBP).	Necessary for the ATP-dependent first step of spliceosome assembly. Binds to the intron branch point sequence (BPS) 5'-UACUAAC-3' of the pre-mRNA. May act as transcription repressor.	3D-structure; Acetylation; Alternative splicing; Direct protein sequencing; Metal-binding; mRNA processing; mRNA splicing; Nucleus; Phosphoprotein; Polymorphism; Repressor; RNA-binding; Spliceosome; Transcription; Transcription regulation; Zinc; Zinc-finger.
	2(3)	2(4)						**KCTD15**	3	11.1	Q96SI1	-	BTB/POZ domain-containing protein KCTD15.		Alternative splicing; Phosphoprotein; Polymorphism.
		2(3)	2(3)					**PLOD1**	3	9.8	Q02809	Endoplasmic reticulum	Procollagen-lysine,2-oxoglutarate 5-dioxygenase 1 precursor (EC 1.14.11.4) (Lysyl hydroxylase 1) (LH1).	Forms hydroxylysine residues in -Xaa-Lys-Gly- sequences in collagens. These hydroxylysines serve as sites of attachment for carbohydrate units and are essential for the stability of the intermolecular collagen cross-links.	Dioxygenase; Direct protein sequencing; Disease mutation; Ehlers-Danlos syndrome; Endoplasmic reticulum; Glycoprotein; Iron; Membrane; Metal-binding; Oxidoreductase; Polymorphism; Signal; Vitamin C.
	3(3)		2(3)					**TBX1**	3	11.1	O43435	Nucleus	T-box transcription factor TBX1 (T-box protein 1) (Testis-specific T- box protein).	Probable transcriptional regulator involved in developmental processes. Is required for normal development of the pharyngeal arch arteries (By similarity).	Alternative splicing; Disease mutation; DNA-binding; Nucleus; Polymorphism; Transcription; Transcription regulation.
		6(6)					2(2)	**RSBN1L**	3	11.8	Q6PCB5	-	Round spermatid basic protein 1-like protein.		Alternative splicing; Nucleus.

					2(2)			**RAD50**	3	5.7	Q92878	Nucleus; Nucleolus	DNA repair protein RAD50 (EC 3.6.-.-) (hRAD50).	Component of the MRN complex, which plays a central role in double-strand break (DSB) repair, DNA recombination, maintenance of telomere integrity and meiosis. The complex possesses single-strand endonuclease activity and double-strand- specific 3'-5' exonuclease activity, which are provided by MRE11A. RAD50 may be required to bind DNA ends and hold them in close proximity. This could facilitate searches for short or long regions of sequence homology in the recombining DNA templates, and may also stimulate the activity of DNA ligases and/or restrict the nuclease activity of MRE11A to prevent nucleolytic degradation past a given point. The complex may also be required for DNA damage signaling via activation of the ATM kinase. In telomeres the MRN complex may modulate t-loop formation.	Alternative splicing; ATP-binding; Cell cycle; Chromosomal protein; Coiled coil; DNA damage; DNA repair; Hydrolase; Meiosis; Metal-binding; Nucleotide-binding; Nucleus; Phosphoprotein; Polymorphism; Telomere; Zinc.
2(3)		2(4)						**TRPC5**	3	1.7	Q9UL62	Plasma membrane	Short transient receptor potential channel 5 (TrpC5) (Htrp-5) (Htrp5).	Thought to form a receptor-activated non-selective calcium permeant cation channel. Probably is operated by a phosphatidylinositol second messenger system activated by receptor tyrosine kinases or G-protein coupled receptors. Has also been shown to be calcium-selective (By similarity). May also be activated by intracellular calcium store depletion.	ANK repeat; Calcium; Calcium channel; Calcium transport; Glycoprotein; Ion transport; Ionic channel; Membrane; Repeat; Transmembrane; Transport.
								**C10orf80**	3	5.3	Q5T655	-	Leucine-rich repeat-containing protein C10orf80.		Coiled coil; Leucine-rich repeat; Polymorphism; Repeat.
3(6)		5(18)	3(3)					**GTF2A2**	3	34.9	P52657	Nucleus	Transcription initiation factor IIA gamma chain (TFIIA P12 subunit) (TFIIA-12) (TFIIAS) (TFIIA-gamma).	TFIIA is a component of the transcription machinery of RNA polymerase II and plays an important role in transcriptional activation. TFIIA in a complex with TBP mediates transcriptional activity.	3D-structure; Nucleus; Transcription; Transcription regulation.
3(5)	3(3)						2(5)	**POLD1**	3	5.9	P28340	Nucleus	DNA polymerase delta catalytic subunit (EC 2.7.7.7) (DNA polymerase subunit delta p125).	Possesses two enzymatic activities: DNA synthesis (polymerase) and an exonucleolytic activity that degrades single stranded DNA in the 3'- to 5'-direction. Required with its accessory proteins (proliferating cell nuclear antigen (PCNA) and replication factor C (RFC) or activator 1) for leading strand synthesis. Also involved in completing Okazaki fragments initiated by the DNA polymerase alpha/primase complex.	DNA replication; DNA-binding; DNA-directed DNA polymerase; Exonuclease; Hydrolase; Metal-binding; Nuclease; Nucleotidyltransferase; Nucleus; Polymorphism; Transferase; Zinc; Zinc-finger.
2(2)			2(2)		2(2)			**MLL2**	3	2.6	O14686	Nucleus	Myeloid/lymphoid or mixed-lineage leukemia protein 2 (ALL1-related protein).	May be involved in transcriptional regulation.	Alternative splicing; Coiled coil; Metal-binding; Nucleus; Phosphoprotein; Polymorphism; Repeat; Transcription; Transcription regulation; Zinc; Zinc-finger.
2(2)	2(2)		3(4)					**SYNE1**	3	0.9	Q8NF91	Golgi apparatus; Sarcoplasmic reticulum; Cytoskeleton; Nucleus	Nesprin-1 (Nuclear envelope spectrin repeat protein 1) (Synaptic nuclear envelope protein 1) (Syne-1) (Myocyte nuclear envelope protein 1) (Myne-1) (Enaptin).	Involved in the maintenance of nuclear organization and structural integrity. Probable anchoring protein which theters the nucleus to the cytoskeleton. Connects nuclei to the cytoskeleton by interacting with the nuclear envelope and with F-actin in the cytoplasm.	Actin-binding; Alternative splicing; Coiled coil; Cytoplasm; Cytoskeleton; Membrane; Nucleus; Phosphoprotein; Polymorphism; Repeat; Structural protein; Transmembrane.
	4(5)		3(3)					**POLDIP3**	3	35.4	Q9BY77	Nucleolus	Polymerase delta-interacting protein 3 (46 kDa DNA polymerase delta interaction protein) (p46).		Alternative splicing; Nucleus; Phosphoprotein; RNA-binding.
	5(7)		8(10)				3(4)	**DKFZp313J1712**	3	40.4	Q69YM7		Putative uncharacterized protein DKFZp313J1712 (Fragment).		
	2(2)	2(3)						**RPS6**	3	27.1	A2A3R5	Ribosome; Nucleolus	Ribosomal protein S6 (Ribosomal protein S6, isoform CRA_a).		Ribosomal protein.

	5(5)	2(2)	2(2)					**CCNL1**	3	14.6	Q9UK58	Nucleus; Cytoplasm	Cyclin-L1 (Cyclin-L).	Transcriptional regulator which participates in regulating the pre-mRNA splicing process. Seems to be involved in the regulation of RNA polymerase II (pol II). Functions in association with cyclin-dependent kinases (CDKs) and has a role in the second step of splicing. May be a candidate proto-oncogene in head and neck squamous cell carcinomas (HNSCC). Inhibited by the CDK-specific inhibitor p21.	Alternative splicing; Cyclin; Nucleus; Phosphoprotein; Proto-oncogene; Repeat; Transcription; Transcription regulation.
	7(7)		8(9)					**OPA1**	3	4.8	O60313	Mitochondrion	Dynamin-like 120 kDa protein, mitochondrial precursor (Optic atrophy protein 1) [Contains: Dynamin-like 120 kDa protein, form S1].	Dynamin-related GTPase required for mitochondrial fusion and regulation of apoptosis. May form a diffusion barrier for proteins stored in mitochondrial cristae. Proteolytic processing in response to intrinsic apoptotic signals may lead to disassembly of OPA1 oligomers and release of the caspase activator cytochrome C (CYCS) into the mitochondrial intermembrane space.	Alternative splicing; Apoptosis; Coiled coil; Deafness; Disease mutation; GTP-binding; Membrane; Mitochondrion; Nucleotide-binding; Phosphoprotein; Polymorphism; Sensory transduction; Transit peptide; Transmembrane; Vision.
	4(5)	2(2)	2(2)					**AP2A2**	3	12.6	O94973	Plasma membrane	AP-2 complex subunit alpha-2 (Adapter-related protein complex 2 alpha- 2 subunit) (Alpha2-adaptin) (Alpha-adaptin C) (Adaptor protein complex AP-2 subunit alpha-2) (Clathrin assembly protein complex 2 alpha-C large chain) (100 kDa coated vesicle protein C) (Plasma membrane adaptor HA2/AP2 adaptin alpha C subunit) (Huntingtin-interacting protein HYPJ).	Adaptins are components of the adaptor complexes which link clathrin to receptors in coated vesicles. Clathrin-associated protein complexes are believed to interact with the cytoplasmic tails of membrane proteins, leading to their selection and concentration. Alpha adaptin is a subunit of the plasma membrane adaptor. Binds polyphosphoinositides (By similarity).	Coated pit; Endocytosis; Lipid-binding; Membrane; Phosphoprotein; Protein transport; Transport.
	4(4)		4(4)				2(2)	**ZNF703**	3	12	Q9H7S9	-	Zinc finger protein 703.	May function as a transcriptional repressor (By similarity).	Alternative splicing; Metal-binding; Nucleus; Repressor; Transcription; Transcription regulation; Zinc; Zinc-finger.
	3(4)		4(6)		2(2)			**G3BP2**	3	17	Q9UN86	Cytoplasm	Ras GTPase-activating protein-binding protein 2 (G3BP-2) (GAP SH3 domain-binding protein 2).	Probable scaffold protein that may be involved in mRNA transport (Potential).	Alternative splicing; Cytoplasm; Methylation; mRNA transport; Phosphoprotein; Polymorphism; RNA-binding; Transport.
	3(4)		6(6)				2(2)	**SEC24B**	3	7.1	O95487	Cytoplasm	Protein transport protein Sec24B (SEC24-related protein B).	Component of the COPII coat, that covers ER-derived vesicles involved in transport from the endoplasmic reticulum to the Golgi apparatus. COPII acts in the cytoplasm to promote the transport of secretory, plasma membrane, and vacuolar proteins from the endoplasmic reticulum to the Golgi complex.	Cytoplasm; Endoplasmic reticulum; ER-Golgi transport; Golgi apparatus; Phosphoprotein; Protein transport; Transport.
	2(2)	6(7)	4(4)					**CCNT2**	3	10.7	O60583	Nucleus	Cyclin-T2 (CycT2).	Regulatory subunit of the cyclin-dependent kinase pair (CDK9/cyclin T) complex, also called positive transcription elongation factor B (P-TEFB), which is proposed to facilitate the transition from abortive to production elongation by phosphorylating the CTD (carboxy-terminal domain) of the large subunit of RNA polymerase II (RNAP II).	3D-structure; Alternative splicing; Cell cycle; Cell division; Coiled coil; Cyclin; Host-virus interaction; Nucleus; Phosphoprotein; Transcription; Transcription regulation.
	2(2)	3(3)	3(4)					**OSBPL11**	3	8.7	Q9BXB4	Cytoplasm	Oxysterol-binding protein-related protein 11 (OSBP-related protein 11) (ORP-11).		3D-structure; Lipid transport; Phosphoprotein; Polymorphism; Transport.

	2(2)	2(2)					2(2)	**SKIV2L2**	3	8.3	P42285	Nucleus; Nucleolus	Superkiller viralicidic activity 2-like 2 (EC 3.6.1.-) (ATP-dependent helicase SKIV2L2).	May be involved in pre-mRNA splicing.	ATP-binding; Helicase; Hydrolase; mRNA processing; mRNA splicing; Nucleotide-binding; Nucleus; Spliceosome.
	2(2)		3(3)			2(2)		**ZGPAT**	3	9.8	Q8N5A5	Nucleus	Zinc finger CCCH-type with G patch domain-containing protein (Zinc finger CCCH domain-containing protein 9) (G patch domain-containing protein 6).		Alternative splicing; Metal-binding; Phosphoprotein; Polymorphism; Zinc; Zinc-finger.
	3(3)	3(3)				2(2)		**CHD7**	3	4.6	Q9P2D1	Nucleus	Chromodomain-helicase-DNA-binding protein 7 (EC 3.6.1.-) (ATP- dependent helicase CHD7) (CHD-7).	Probable transcription regulator.	3D-structure; Alternative splicing; ATP-binding; Chromatin regulator; Coiled coil; Disease mutation; DNA-binding; Helicase; Hydrolase; Nucleotide-binding; Nucleus; Phosphoprotein; Polymorphism; Repeat; Transcription; Transcription regulation.
	2(2)	6(7)	3(3)					**KIAA1429**	3	9.9	Q69YN4	-	Protein virilizer homolog.	May be involved in mRNA splicing regulation (By similarity).	Alternative splicing; mRNA processing; mRNA splicing; Nucleus; Phosphoprotein; Polymorphism.
	2(2)	2(2)				2(3)		**MACF1**	3	0.9	Q96PK2	Cytoplasm	Microtubule-actin cross-linking factor 1, isoform 4.	May play a role in cross-linking cytoskeletal proteins by binding intermediate filaments to the N-terminal plectin repeats and microtubules to the C-terminus.	Alternative splicing; Calcium; Coiled coil; Cytoplasm; Cytoskeleton; Phosphoprotein; Repeat.
					2(2)			**DDX3Y**	3	5.3	O15523	Nucleus; Cytoplasm	ATP-dependent RNA helicase DDX3Y (EC 3.6.1.-) (DEAD box protein 3, Y- chromosomal).	Probable ATP-dependent RNA helicase. May play a role in spermatogenesis.	ATP-binding; Cytoplasm; DNA-binding; Helicase; Hydrolase; Nucleotide-binding; Nucleus; Phosphoprotein; RNA-binding.
		3(3)	2(3)		2(2)			**SMC3**	3	8.5	Q9UQE7		Structural maintenance of chromosomes protein 3 (Chondroitin sulfate proteoglycan 6) (Chromosome-associated polypeptide) (hCAP) (Bamacan) (Basement membrane-associated chondroitin proteoglycan).	Involved in chromosome cohesion during cell cycle and in DNA repair. Central component of cohesin complex. The cohesin complex is required for the cohesion of sister chromatids after DNA replication. The cohesin complex apparently forms a large proteinaceous ring within which sister chromatids can be trapped. At anaphase, the complex is cleaved and dissociates from chromatin, allowing sister chromatids to segregate. The cohesin complex may also play a role in spindle pole assembly during mitosis and in chromosome movement.	ATP-binding; Cell cycle; Cell division; Chromosome partition; Coiled coil; Disease mutation; DNA damage; DNA repair; Meiosis; Mitosis; Nucleotide-binding; Nucleus; Phosphoprotein.
		2(2)			2(4)		3(4)	**DHX57**	3	7.8	Q6P158	-	Putative ATP-dependent RNA helicase DHX57 (EC 3.6.1.-) (DEAH box protein 57).	Probable ATP-binding RNA helicase.	Alternative splicing; ATP-binding; Coiled coil; Helicase; Hydrolase; Metal-binding; Nucleotide-binding; Phosphoprotein; Polymorphism; Zinc; Zinc-finger.
					2(3)	2(7)	2(2)	**KIAA1033**	3	3.2	Q2M389	-	Uncharacterized protein KIAA1033.		Alternative splicing; Coiled coil; Polymorphism.
		2(2)			2(2)		3(4)	**MYH13**	3	3.1	Q9UKX3	Cytoplasm	Myosin-13 (Myosin heavy chain 13) (Myosin heavy chain, skeletal muscle, extraocular) (MyHC-eo).	Muscle contraction.	Actin-binding; ATP-binding; Calmodulin-binding; Coiled coil; Cytoplasm; Methylation; Motor protein; Muscle protein; Myosin; Nucleotide-binding; Polymorphism; Thick filament.
		5(5)	2(2)			2(2)		**C20orf27**	3	31.8	Q9GZN8	-	Uncharacterized protein C20orf27.		Direct protein sequencing.

						11(27)		**HSPA1A**	3	74.1	P08107	Cytoplasm; Nucleolus	Heat shock 70 kDa protein 1 (HSP70.1) (HSP70-1/HSP70-2).	In cooperation with other chaperones, Hsp70s stabilize preexistent proteins against aggregation and mediate the folding of newly translated polypeptides in the cytosol as well as within organelles. These chaperones participate in all these processes through their ability to recognize nonnative conformations of other proteins. They bind extended peptide segments with a net hydrophobic character exposed by polypeptides during translation and membrane translocation, or following stress-induced damage.	3D-structure; ATP-binding; Chaperone; Direct protein sequencing; Nucleotide-binding; Phosphoprotein; Polymorphism; Stress response.
						25(92)		**SFPQ**	3	57.3	P23246	Nucleus; Nucleolus	Splicing factor, proline- and glutamine-rich (Polypyrimidine tract- binding protein-associated-splicing factor) (PTB-associated-splicing factor) (PSF) (DNA-binding p52/p100 complex, 100 kDa subunit) (100 kDa DNA-pairing protein) (hPOMp100).	DNA- and RNA binding protein, involved in several nuclear processes. Essential pre-mRNA splicing factor required early in spliceosome formation and for splicing catalytic step II, probably as an heteromer with NONO. Binds to pre-mRNA in spliceosome C complex, and specifically binds to intronic polypyrimidine tracts. Interacts with U5 snRNA, probably by binding to a purine-rich sequence located on the 3' side of U5 snRNA stem 1b. May be involved in a pre-mRNA coupled splicing and polyadenylation process as component of a snRNP-free complex with SNRPA/U1A. The SFPQ-NONO heteromer associated with MATR3 may play a role in nuclear retention of defective RNAs. SFPQ may be involved in homologous DNA pairing; in vitro, promotes the invasion of ssDNA between a duplex DNA and produces a D-loop formation. The SFPQ-NONO heteromer may be involved in DNA unwinding by modulating the function of topoisomerase I/TOP1; in vitro, stimulates dissociation of TOP1 from DNA after cleavage and enhances its jumping between separate DNA helices. The SFPQ-NONO heteromer may be involved in DNA nonhomologous end joining (NHEJ) required for double-strand break repair and V(D)J recombination and may stabilize paired DNA ends; in vitro, the complex strongly stimulates DNA end joining, binds directly to the DNA substrates and cooperates with the Ku70/G22P1-Ku80/XRCC5 (Ku) dimer to establish a functional preligation complex. SFPQ is involved in transcriptional regulation. Transcriptional repression is probably mediated by an interaction of SFPQ with SIN3A and subsequent recruitment of histone deacetylases (HDACs). The SFPQ-NONO/SF-1 complex binds to the CYP17 promoter and regulates basal and cAMP- dependent transcriptional avtivity. SFPQ isoform Long binds to the DNA binding domains (DBD) of nuclear hormone receptors, like RXRA and probably THRA, and acts as transcriptional corepressor in absence of hormone ligands. Binds the DNA sequence 5'-CTGAGTC-3' in the insulin-like growth	Activator; Alternative splicing; Chromosomal rearrangement; Direct protein sequencing; DNA damage; DNA recombination; DNA repair; DNA-binding; Methylation; mRNA processing; mRNA splicing; Nucleus; Phosphoprotein; Repeat; Repressor; RNA-binding; Transcription; Transcription regulation.
						2(5)		**TUBB1**	3	5.5	Q9H4B7	Nucleolus	Tubulin beta-1 chain.	Tubulin is the major constituent of microtubules. It binds two moles of GTP, one at an exchangeable site on the beta chain and one at a non-exchangeable site on the alpha-chain (By similarity).	Direct protein sequencing; GTP-binding; Microtubule; Nucleotide-binding; Polymorphism.

			2(2)					**PCMT1**	3	37.3	P22061	Cytoplasm	Protein-L-isoaspartate(D-aspartate) O-methyltransferase (EC 2.1.1.77) (Protein-beta-aspartate methyltransferase) (PIMT) (Protein L- isoaspartyl/D-aspartyl methyltransferase) (L-isoaspartyl protein carboxyl methyltransferase).	Catalyzes the methyl esterification of L-isoaspartyl and D-aspartyl residues in peptides and proteins that result from spontaneous decomposition of normal L-aspartyl and L-asparaginyl residues. It plays a role in the repair and/or degradation of damaged proteins. Acts on microtubule-associated protein 2, calreticulin, clathrin light chains a and b, Ubiquitin carboxyl- terminal hydrolase isozyme L1, phosphatidylethanolamine-binding protein 1, stathmin, beta-synuclein and alpha-synuclein (By similarity).	3D-structure; Acetylation; Alternative splicing; Cytoplasm; Direct protein sequencing; Methyltransferase; Polymorphism; S-adenosyl-L-methionine; Transferase.	
			3(3)					**GANAB**	2	6.5	Q14697	Endoplasmic reticulum	Neutral alpha-glucosidase AB precursor (EC 3.2.1.84) (Glucosidase II subunit alpha).	Cleaves sequentially the 2 innermost alpha-1,3-linked glucose residues from the Glc(2)Man(9)GlcNAc(2) oligosaccharide precursor of immature glycoproteins.	Alternative splicing; Direct protein sequencing; Endoplasmic reticulum; Glycoprotein; Golgi apparatus; Hydrolase; Polymorphism; Signal.	
			2(3)					**PRMT5**	2	8.4	A8MZ91	Cytoplasm	Uncharacterized protein PRMT5 (Protein arginine methyltransferase 5, isoform CRA_d).		Methyltransferase; Transferase.	
			2(2)					**KIAA2022**	2	3.7	Q5QGS0		Uncharacterized protein KIAA2022.		Chromosomal rearrangement.	
		2(2)						**TDRD6**	2	1.6	O60522	-	Tudor domain-containing protein 6 (Antigen NY-CO-45) (Cancer/testis antigen 41.2) (CT41.2).		Polymorphism; Repeat.	
3(4)								**SYNE2**	2	0.7	Q8WXH0	Sarcoplasmic reticulum; Plasma membrane; Nucleus; Nucleolus; Cytoplasm	Nesprin-2 (Nuclear envelope spectrin repeat protein 2) (Syne-2) (Synaptic nuclear envelope protein 2) (Nucleus and actin connecting element protein) (Protein NUANCE).	Involved in the maintenance of nuclear organization and structural integrity. Probable anchoring protein which theters the nucleus to the cytoskeleton. Connects nuclei to the cytoskeleton by interacting with the nuclear envelope and with F-actin in the cytoplasm.	Actin-binding; Alternative splicing; Coiled coil; Cytoplasm; Cytoskeleton; Leucine-rich repeat; Membrane; Nucleus; Phosphoprotein; Polymorphism; Repeat; Structural protein; Transmembrane.	
		2(2)						**CENPF**	2	2.3	P49454	Nucleus	Centromere protein F (Kinetochore protein CENP-F) (Mitosin) (AH antigen).	Probably required for kinetochore function, involved in chromosome segregation during mitosis. Interacts with retinoblastoma protein (RB), CENP-E and BUBR1.	Cell cycle; Cell division; Centromere; Chromosomal protein; Coiled coil; Lipoprotein; Mitosis; Nucleus; Phosphoprotein; Polymorphism; Prenylation; Repeat.	
			2(2)					**TAF2**	2	6.5	Q6P1X5	Nucleus	Transcription initiation factor TFIID subunit 2 (Transcription initiation factor TFIID 150 kDa subunit) (TBP-associated factor 150 kDa) (TAFII-150) (TAFII150) (150 kDa cofactor of initiator function) (RNA polymerase II TBP-associated factor subunit B).	Transcription factor TFIID is one of the general factors required for accurate and regulated initiation by RNA polymerase II. TFIID is a multimeric protein complex that plays a central role in mediating promoter responses to various activators and repressors. It requires core promoter-specific cofactors for productive transcription stimulation. TAF2 stabilizes TFIID binding to core promoter.	Nucleus; Phosphoprotein; Polymorphism; Transcription; Transcription regulation.	
	2(2)							**RYR2**	2	1.4	Q92736	Sarcoplasmic reticulum; Endoplasmic reticulum	Ryanodine receptor 2 (Cardiac muscle-type ryanodine receptor) (RyR2) (RYR-2) (Cardiac muscle ryanodine receptor-calcium release channel) (hRYR-2).	Communication between transverse-tubules and sarcoplasmic reticulum. Contraction of cardiac muscle is triggered by release of calcium ions from SR following depolarization of T- tubules (By similarity).	Alternative splicing; Calcium; Calcium channel; Calcium transport; Calmodulin-binding; Cardiomyopathy; Disease mutation; Glycoprotein; Ion transport; Ionic channel; Membrane; Phosphoprotein; Polymorphism; Receptor; Repeat; Transmembrane; Transport.	
							2(3)	**RPS10**	2	31.5	P46783	Ribosome; Nucleolus	40S ribosomal protein S10.		Cytoplasm; Direct protein sequencing; Phosphoprotein; Ribonucleoprotein; Ribosomal protein.	
		2(3)						**ATN1**	2	5.9	P54259	Cytoplasm; Nucleus	Atrophin-1 (Dentatorubral-pallidoluysian atrophy protein).		Epilepsy; Neurodegeneration; Phosphoprotein; Polymorphism; Triplet repeat expansion.	

3(3)								**CDC37**	2	13	Q16543	Cytoplasm	Hsp90 co-chaperone Cdc37 (Hsp90 chaperone protein kinase-targeting subunit) (p50Cdc37).	Co-chaperone that binds to numerous kinases and promotes their interaction with the Hsp90 complex, resulting in stabilization and promotion of their activity.	3D-structure; Chaperone; Cytoplasm; Phosphoprotein; Polymorphism.
						2(2)		**ARHGEF18**	2	6.2	O60274	-	KIAA0521 protein (Fragment).		
			3(3)					**CDC2L5**	2	3.4	Q14004	Nucleus; Nucleolus	Cell division cycle 2-like protein kinase 5 (EC 2.7.11.22) (CDC2-related protein kinase 5) (Cholinesterase-related cell division controller).	May be a controller of the mitotic cell cycle. Involved in the blood cell development.	Alternative splicing; ATP-binding; Kinase; Nucleotide-binding; Phosphoprotein; Polymorphism; Serine/threonine-protein kinase; Transferase.
							3(5)	**P140**	2	3.2	Q9C0H9		p130Cas-associated protein (p140Cap) (SNAP-25-interacting protein) (SNIP).	Delays the onset of cell spreading in the early stages of cell adhesion to fibronectin. Also involved in calcium-dependent exocytosis from PC12 cells.	Alternative splicing; Coiled coil; Cytoplasm; Cytoskeleton; Direct protein sequencing; Exocytosis; Phosphoprotein.
2(2)								**RNF40**	2	6.7	O75150	Cytoplasm; Integral to membrane; Nucleus	E3 ubiquitin-protein ligase BRE1B (EC 6.3.2.-) (BRE1-B) (RING finger protein 40) (95 kDa retinoblastoma-associated protein) (RBP95).	E3 ubiquitin-protein ligase that mediates monoubiquitination of 'Lys-120' of histone H2B. H2B 'Lys-120' ubiquitination gives a specific tag for epigenetic transcriptional activation and is also prerequisite for histone H3 'Lys-4' and 'Lys-79' methylation. Forms a ubiquitin ligase complex in cooperation with the E2 enzyme UBE2E1/UBCH6. It thereby plays a central role in histone code and gene regulation. Required for transcriptional activation of Hox genes.	Alternative splicing; Chromatin regulator; Chromosomal protein; Coiled coil; Ligase; Metal-binding; Nucleus; Phosphoprotein; Ubl conjugation pathway; Zinc; Zinc-finger.
		2(3)						**UTRN**	2	2	P46939	Cytoplasm; Plasma membrane	Utrophin (Dystrophin-related protein 1) (DRP1) (DRP).	May play a role in anchoring the cytoskeleton to the plasma membrane (By similarity).	3D-structure; Actin-binding; Calcium; Cell junction; Cytoplasm; Cytoskeleton; Membrane; Metal-binding; Phosphoprotein; Postsynaptic cell membrane; Repeat; Structural protein; Synapse; Zinc; Zinc-finger.
							2(2)	**HK1**	2	3.1	P19367	Cytoplasm; Mitochondrial membrane; Plasma membrane	Hexokinase-1 (EC 2.7.1.1) (Hexokinase type I) (HK I) (Brain form hexokinase).		3D-structure; Allosteric enzyme; Alternative splicing; ATP-binding; Direct protein sequencing; Disease mutation; Glycolysis; Kinase; Membrane; Mitochondrion; Nucleotide-binding; Polymorphism; Repeat; Transferase.
			2(2)					**DMD**	2	1.7	P11532	Cytoplasm	Dystrophin.	May play a role in anchoring the cytoskeleton to the plasma membrane.	3D-structure; Actin-binding; Alternative splicing; Calcium; Cardiomyopathy; Cytoplasm; Cytoskeleton; Disease mutation; Membrane; Metal-binding; Phosphoprotein; Polymorphism; Repeat; Structural protein; Zinc; Zinc-finger.
			2(2)					**GARNL1**	2	2.6	Q6GYQ0	Nucleus; Cytoplasm	GTPase-activating Rap/Ran-GAP domain-like 1 (GAP-related-interacting partner to E12) (GRIPE) (Tuberin-like protein 1).	Interacting partner of the transcription factor TCF3/isoform E12, mainly in the developing embryonic forebrain. May be an important transcriptional regulator of downstream target genes under the control of TCF3/E12, by disrupting HLH dimer formation of TCF3/E12 with other proteins. May be involved in neuronal differentiation (By similarity).	Alternative splicing; Coiled coil; Cytoplasm; GTPase activation; Nucleus; Phosphoprotein; Polymorphism.

2(2)	3(4)							**DNAJA2**	2	21.4	O60884	Cytoplasm; Nucleus; Mitochondrion; Microsome	DnaJ homolog subfamily A member 2 (HIRA-interacting protein 4) (Cell cycle progression restoration gene 3 protein) (Dnj3) (Renal carcinoma antigen NY-REN-14).	Co-chaperone of Hsc70.	Chaperone; Lipoprotein; Membrane; Metal-binding; Phosphoprotein; Prenylation; Repeat; Zinc; Zinc-finger.
2(2)					2(2)			**SNW1**	2	14.2	Q13573	Nucleus; Nucleolus	SNW domain-containing protein 1 (Nuclear protein SkiP) (Ski-interacting protein) (Nuclear receptor coactivator NCoA-62).	Involved in vitamin D-mediated transcription. Can function as a splicing factor in pre-mRNA splicing.	mRNA processing; mRNA splicing; Nucleus; Phosphoprotein; Spliceosome.
2(3)		5(12)						**GTF2A1**	2	13.6	P52655	Nucleus	Transcription initiation factor IIA subunit 1 (General transcription factor IIA1) (TFIIA-42) (TFIIAL) [Contains: Transcription initiation factor IIA alpha chain (TFIIA p35 subunit); Transcription initiation factor IIA beta chain (TFIIA p19 subunit)].	TFIIA is a component of the transcription machinery of RNA polymerase II and plays an important role in transcriptional activation. TFIIA in a complex with TBP mediates transcriptional activity.	3D-structure; Alternative initiation; Direct protein sequencing; Nucleus; Phosphoprotein; Polymorphism; Transcription; Transcription regulation.
2(2)		2(2)						**DHX33**	2	5	Q9H6R0	Nucleolus	Putative ATP-dependent RNA helicase DHX33 (EC 3.6.1.-) (DEAH box protein 33).		Alternative splicing; ATP-binding; Direct protein sequencing; Helicase; Hydrolase; Nucleotide-binding; Nucleus; Phosphoprotein.
2(2)			3(3)					**KIF13B**	2	1.9	Q9NQT8	Cytoplasm	Kinesin-like protein KIF13B (Kinesin-like protein GAKIN).	May be involved in reorganization of the cortical cytoskeleton. May be functionally important for the intracellular trafficking of MAGUKs and associated protein complexes.	3D-structure; ATP-binding; Coiled coil; Cytoplasm; Cytoskeleton; Microtubule; Motor protein; Nucleotide-binding; Phosphoprotein.
2(3)		2(4)						**ARHGAP20**	2	1.6	Q9P2F6	-	Rho GTPase-activating protein 20 (Rho-type GTPase-activating protein 20).	GTPase activator for the Rho-type GTPases by converting them to an inactive GDP-bound state (By similarity).	Alternative splicing; Anti-oncogene; Cell cycle; Chromosomal rearrangement; GTPase activation; Polymorphism.
2(3)		2(4)						**MCM3AP**	2	0.6	O60318	Nucleus; Cytoplasm	80 kDa MCM3-associated protein (Protein GANP).	May be involved in the nuclear localization pathway of MCM3.	Cytoplasm; Nucleus; Polymorphism.
	8(9)		6(7)					**RAVER1**	2	16.2	Q8IY67		Ribonucleoprotein PTB-binding 1 (Protein raver-1).	Cooperates with PTBP1 to modulate regulated alternative splicing events. Promotes exon skipping. Cooperates with PTBP1 to modulate switching between mutually exclusive exons during maturation of the TPM1 pre-mRNA (By similarity).	Alternative splicing; Cytoplasm; Nucleus; Phosphoprotein; Repeat; RNA-binding.
	6(6)		7(9)					**EDC3**	2	25.6	Q96F86		Enhancer of mRNA-decapping protein 3 (YjeF domain-containing protein 1) (LSM16 homolog).	In the process of mRNA degradation, may play a role in mRNA decapping.	Cytoplasm; Phosphoprotein.
	4(4)		6(6)					**U2AF1L4**	2	22.8	Q8WU68	Nucleus	Splicing factor U2AF 26 kDa subunit (U2 small nuclear RNA auxiliary factor 1-like protein 4) (U2 small nuclear RNA auxiliary factor 1-like protein 3) (U2(RNU2) small nuclear RNA auxiliary factor 1-like protein 3) (U2AF1-like protein 3).	RNA-binding protein that function as a pre-mRNA splicing factor. Plays a critical role in both constitutive and enhancer- dependent splicing by mediating protein-protein interactions and protein-RNA interactions required for accurate 3'-splice site selection. Acts by enhancing the binding of U2AF2 to weak pyrimidine tracts. Also participates in the regulation of alternative pre-mRNA splicing. Activates exon 5 skipping of PTPRC during T cell activation; an event reversed by GFI1. Binds to RNA at the AG dinucleotide at the 3'-splice site (By similarity).	Alternative splicing; Metal-binding; mRNA processing; mRNA splicing; Nucleus; Repeat; RNA-binding; Spliceosome; Zinc; Zinc-finger.
	8(17)		10(16)					**U2AF2**	2	24.3	A6NN86	Nucleus; Cytoplasm; Nucleolus	Uncharacterized protein U2AF2.		

	2(5)		3(5)					**mer5**	2	22.7	Q14579		Humer (Fragment).		
	2(5)		3(5)					**PRDX3**	2	21.1	P30048	Mitochondrion	Thioredoxin-dependent peroxide reductase, mitochondrial precursor (EC 1.11.1.15) (Peroxiredoxin-3) (PRX III) (Antioxidant protein 1) (AOP-1) (Protein MER5 homolog) (HBC189).	Involved in redox regulation of the cell. Protects radical-sensitive enzymes from oxidative damage by a radical-generating system. Acts synergistically with MAP3K13 to regulate the activation of NF-kappa-B in the cytosol.	Antioxidant; Direct protein sequencing; Mitochondrion; Oxidoreductase; Peroxidase; Redox-active center; Transit peptide.
	4(5)		6(7)					**IVD**	2	17.3	P26440	Mitochondrion	Isovaleryl-CoA dehydrogenase, mitochondrial precursor (EC 1.3.99.10) (IVD).		3D-structure; Acetylation; Direct protein sequencing; Disease mutation; FAD; Flavoprotein; Mitochondrion; Oxidoreductase; Transit peptide.
	3(3)		3(3)					**FHL1**	2	17.1	Q13642	Cytoplasm	Four and a half LIM domains protein 1 (FHL-1) (Skeletal muscle LIM- protein 1) (SLIM 1) (SLIM).	May have an involvement in muscle development or hypertrophy.	3D-structure; Alternative splicing; Cytoplasm; Developmental protein; Differentiation; Direct protein sequencing; LIM domain; Metal-binding; Nucleus; Repeat; Zinc; Zinc-finger.
	1(11)		1(14)					**LDOC1L**	2	10.5	Q6ICC9	-	Protein LDOC1L (Leucine zipper protein down-regulated in cancer cells- like) (Mammalian retrotransposon-derived protein 6).		Coiled coil.
	3(3)		3(3)					**ARMC6**	2	9.4	Q6NXE6	-	Armadillo repeat-containing protein 6.		Phosphoprotein; Repeat.
	2(2)		2(2)					**DKFZp686M13204**	2	17.9	Q5HYN2		Putative uncharacterized protein DKFZp686M13204 (Fragment).		
	2(2)		2(2)					**SFRS11**	2	15.7	Q8IWE6	Nucleus	Splicing factor, arginine/serine-rich 11 (Splicing factor, arginine/serine-rich 11, isoform CRA_b).		
	2(2)		2(2)					**SNRP70**	2	7.6	P08621	Nucleus	U1 small nuclear ribonucleoprotein 70 kDa (U1 snRNP 70 kDa) (snRNP70) (U1-70K).	Mediates the splicing of pre-mRNA by binding to the loop I region of U1-snRNA. The truncated isoforms cannot bind U1-snRNA.	Alternative splicing; Direct protein sequencing; mRNA processing; Nucleus; Phosphoprotein; Ribonucleoprotein; RNA-binding.
	3(3)		3(4)					**MARK3**	2	7.8	P27448	Cytoplasm	MAP/microtubule affinity-regulating kinase 3 (EC 2.7.11.1) (Cdc25C- associated protein kinase 1) (cTAK1) (C-TAK1) (Serine/threonine protein kinase p78) (Ser/Thr protein kinase PAR-1) (Protein kinase STK10).	Involved in the specific phosphorylation of microtubule-associated proteins for tau, MAP2 and MAP4. Phosphorylates CDC25C on 'Ser-216'.	Alternative splicing; ATP-binding; Kinase; Nucleotide-binding; Phosphoprotein; Serine/threonine-protein kinase; Transferase.
	3(3)		4(4)					**PFKL**	2	8.5	P17858	Cytoplasm	6-phosphofructokinase, liver type (EC 2.7.1.11) (Phosphofructokinase 1) (Phosphohexokinase) (Phosphofructo-1-kinase isozyme B) (PFK-B).		Allosteric enzyme; Alternative splicing; ATP-binding; Direct protein sequencing; Glycolysis; Kinase; Magnesium; Metal-binding; Nucleotide-binding; Phosphoprotein; Polymorphism; Repeat; Transferase.
	3(3)		3(3)					**SFRS16**	2	7.6	Q8N2M8	Nucleus	Splicing factor, arginine/serine-rich 16 (Suppressor of white-apricot homolog 2).	Probably functions as an alternative splicing regulator. May regulate the mRNA splicing of genes such as CLK1. May act by regulating members of the CLK kinase family (By similarity).	Alternative splicing; mRNA processing; mRNA splicing; Nucleus; Phosphoprotein; Polymorphism.

	2(2)		3(3)					**DKC1**	2	9.7	O60832	Nucleus; Nucleolus	H/ACA ribonucleoprotein complex subunit 4 (EC 5.4.99.-) (Dyskerin) (Nucleolar protein family A member 4) (snoRNP protein DKC1) (Nopp140-associated protein of 57 kDa) (Nucleolar protein NAP57) (CBF5 homolog).	Required for ribosome biogenesis and telomere maintenance. Probable catalytic subunit of H/ACA small nucleolar ribonucleoprotein (H/ACA snoRNP) complex, which catalyzes pseudouridylation of rRNA. This involves the isomerization of uridine such that the ribose is subsequently attached to C5, instead of the normal N1. Each rRNA can contain up to 100 pseudouridine ('psi') residues, which may serve to stabilize the conformation of rRNAs. Also required for correct processing or intranuclear trafficking of TERC, the RNA component of the telomerase reverse transcriptase (TERT) holoenzyme.	Acetylation; Direct protein sequencing; Disease mutation; Isomerase; Nucleus; Phosphoprotein; Ribonucleoprotein; Ribosome biogenesis; RNA-binding; rRNA processing.
	2(2)		3(3)					**FXR2**	2	10.5	P51116	Cytoplasm; Ribosome; Nucleolus; Nucleus	Fragile X mental retardation syndrome-related protein 2.	RNA-binding protein.	Cytoplasm; Phosphoprotein; Repeat; RNA-binding.
	3(3)		2(2)					**DARS2**	2	8.1	Q6PI48	-	Aspartyl-tRNA synthetase, mitochondrial precursor (EC 6.1.1.12) (Aspartate--tRNA ligase) (AspRS).		Aminoacyl-tRNA synthetase; ATP-binding; Disease mutation; Ligase; Mitochondrion; Nucleotide-binding; Polymorphism; Protein biosynthesis; Transit peptide.
	2(2)		2(2)					**SDF2L1**	2	6	Q9HCN8	Endoplasmic reticulum	Stromal cell-derived factor 2-like protein 1 precursor (SDF2-like protein 1) (PWP1-interacting protein 8).		Endoplasmic reticulum; Repeat; Signal.
	2(2)		3(3)					**TBC1D15**	2	9.3	Q8TC07	-	TBC1 domain family member 15.	May act as a GTPase-activating protein for Rab family protein(s).	Alternative splicing; GTPase activation; Phosphoprotein.
	2(2)		3(3)					**CLPB**	2	7.8	Q9H078	-	Caseinolytic peptidase B protein homolog (Suppressor of potassium transport defect 3).	May function as a regulatory ATPase and be related to secretion/protein trafficking process.	Alternative splicing; ANK repeat; ATP-binding; Nucleotide-binding; Repeat.
	2(2)		3(3)					**USP15**	2	7.2	Q9Y4E8	Cytoplasm; Nucleolus	Ubiquitin carboxyl-terminal hydrolase 15 (EC 3.1.2.15) (Ubiquitin thioesterase 15) (Ubiquitin-specific-processing protease 15) (Deubiquitinating enzyme 15) (Unph-2) (Unph4).		3D-structure; Alternative splicing; Hydrolase; Phosphoprotein; Protease; Thiol protease; Ubl conjugation pathway.
	2(2)	2(2)						**GCFC**	2	12.3	Q9Y5B6		GC-rich sequence DNA-binding factor homolog.	Possible transcription factor.	Alternative splicing; DNA-binding; Nucleus; Phosphoprotein; Transcription; Transcription regulation.
	3(4)		3(3)					**HSPA4L**	2	7.3	O95757	Cytoplasm	Heat shock 70 kDa protein 4L (Osmotic stress protein 94) (Heat shock 70-related protein APG-1).	Possesses chaperone activity in vitro where it inhibits aggregation of citrate synthase (By similarity).	ATP-binding; Chaperone; Cytoplasm; Nucleotide-binding; Nucleus; Phosphoprotein; Polymorphism; Stress response.
	2(2)	4(4)						**REPIN1**	2	11.3	Q9BWE0	Nucleus	Replication initiator 1 (Zinc finger protein 464) (DHFR oribeta- binding protein RIP60) (60 kDa replication initiation region protein) (60 kDa origin-specific DNA-binding protein) (ATT-binding protein).	Sequence-specific double-stranded DNA-binding protein required for initiation of chromosomal DNA replication. Binds on 5'-ATT-3' reiterated sequences downstream of the origin of bidirectional replication (OBR) and a second, homologous ATT sequence of opposite orientation situated within the OBR zone. Facilitates DNA bending.	Direct protein sequencing; DNA replication; DNA-binding; Metal-binding; Nucleus; Polymorphism; Repeat; Zinc; Zinc-finger.
	2(2)		2(2)					**DHX40**	2	3.7	Q8IX18	Nucleus	Probable ATP-dependent RNA helicase DHX40 (EC 3.6.1.-) (DEAH box protein 40) (Protein PAD).	Probable ATP-dependent RNA helicase (By similarity).	Alternative splicing; ATP-binding; Helicase; Hydrolase; Nucleotide-binding.

	2(2)		3(3)					**LLGL1**	2	3.4	A6NCT0	Cytoplasm	Uncharacterized protein LLGL1.		WD repeat.
	2(2)		2(2)					**MYOM2**	2	4.1	P54296	Cytoplasm	Myomesin-2 (M-protein) (165 kDa titin-associated protein) (165 kDa connectin-associated protein).	Major component of the vertebrate myofibrillar M band. Binds myosin, titin, and light meromyosin. This binding is dose dependent.	Immunoglobulin domain; Muscle protein; Polymorphism; Repeat; Thick filament.
	2(2)	3(3)						**BRD2**	2	5.2	P25440	Nucleus; Cytoplasm	Bromodomain-containing protein 2 (Protein RING3) (O27.1.1).	May play a role in spermatogenesis or folliculogenesis (By similarity).	3D-structure; Alternative splicing; Bromodomain; Nucleus; Phosphoprotein; Polymorphism; Repeat.
	2(2)	2(2)						**LKAP**	2	4.4	Q9Y4F3		Limkain-b1.		Alternative splicing; Peroxisome; Phosphoprotein; RNA-binding.
	2(2)	4(5)						**ZC3H13**	2	2.1	Q5T200	-	Zinc finger CCCH domain-containing protein 13.		Alternative splicing; Coiled coil; Metal-binding; Phosphoprotein; Polymorphism; Repeat; Zinc; Zinc-finger.
	2(2)		2(2)					**NFRKB**	2	1.6	Q6P4R8	Nucleus	Nuclear factor related to kappa-B-binding protein (DNA-binding protein R kappa-B).	Binds to the DNA consensus sequence 5'-GGGGAATCTCC-3'.	Alternative splicing; DNA-binding; Nucleus; Phosphoprotein.
	2(3)		2(3)					**DHX37**	2	1.6	Q8IY37	Nucleolus	Probable ATP-dependent RNA helicase DHX37 (EC 3.6.1.-) (DEAH box protein 37).		ATP-binding; Helicase; Hydrolase; Nucleotide-binding.
	2(3)		2(4)					**REV3L**	2	2.7	O60673	Nucleus	DNA polymerase zeta catalytic subunit (EC 2.7.7.7) (hREV3).		Alternative splicing; DNA damage; DNA repair; DNA replication; DNA-binding; DNA-directed DNA polymerase; Metal-binding; [illegible]
	2(2)		2(2)					**SRRM2**	2	2.2	Q9UQ35	Nucleus	Serine/arginine repetitive matrix protein 2 (Serine/arginine-rich splicing factor-related nuclear matrix protein of 300 kDa) (Ser/Arg- related nuclear matrix protein) (SR-related nuclear matrix protein of 300 kDa) (Splicing coactivator subunit SRm300) (300 kDa nuclear matrix antigen).	Part of pre- and post-splicing multiprotein mRNP complexes. May be involved in pre-mRNA processing events. Binds to RNA.	Alternative splicing; Coiled coil; Direct protein sequencing; mRNA processing; mRNA splicing; Nucleus; Phosphoprotein; Polymorphism; RNA-binding; Spliceosome.
					2(2)		2(2)	**CDK5R2**	2	8.7	Q13319		Extracellular; Cytoplasm; Plasma membrane	Cyclin-dependent kinase 5 activator 2 precursor (CDK5 activator 2) (Cyclin-dependent kinase 5 regulatory subunit 2) (P39) (P39I).	Activator of CDK5/TPKII.
					2(7)		2(2)	**CAPS**	2	5.8	Q13938	Cytoplasm	Calcyphosin (Calcyphosine).	May play a role in the regulation of ionic transport. Binds calcium.	Calcium; Cytoplasm; Phosphoprotein; Repeat.
					2(7)		2(2)	**FLJ00390**	2	4	**Q8NF12**		FLJ00390 protein (Fragment).		
					2(4)	2(4)		**STXBP4**	2	4.3	Q6ZWJ1	-	Syntaxin-binding protein 4 (Syntaxin 4-interacting protein) (STX4- interacting protein) (Synip).	Plays a role in the translocation of transport vesicles from the cytoplasm to the plasma membrane. Inhibits the translocation of SLC2A4 from intracellular vesicles to the plasma membrane by STX4A binding and preventing the interaction between STX4A and VAMP2. Stimulation with insulin disrupts the interaction with STX4A, leading to increased levels of SLC2A4 at the plasma membrane. May also play a role in the regulation of insulin release by pancreatic beta cells after stimulation by glucose (By similarity).	Alternative splicing; Coiled coil; Cytoplasm; Phosphoprotein; Repeat.
					2(2)	2(2)		**KIF7**	2	8.7	Q2M1P5	-	Kinesin-like protein KIF7.		ATP-binding; Coiled coil; Motor protein; Nucleotide-binding; Polymorphism.

					2(3)	3(4)		**HD**	2	1.3	P42858	Cytoplasm; Nucleus; Plasma membrane; Golgi apparatus	Huntingtin (Huntington disease protein) (HD protein).	May play a role in microtubule-mediated transport or vesicle function.	3D-structure; Apoptosis; Cytoplasm; Disease mutation; Nucleus; Phosphoprotein; Polymorphism; Repeat; Triplet repeat expansion; Ubl conjugation.
		2(2)		2(2)				**MECP2**	2	10.9	P51608	Nucleus	Methyl-CpG-binding protein 2 (MeCP-2 protein) (MeCP2).	Chromosomal protein that binds to methylated DNA. It can bind specifically to a single methyl-CpG pair. It is not influenced by sequences flanking the methyl-CpGs. Mediates transcriptional repression through interaction with histone deacetylase and the corepressor SIN3A.	3D-structure; Alternative splicing; Chromosomal rearrangement; Disease mutation; DNA-binding; Nucleus; Phosphoprotein; Polymorphism; Repeat; Repressor; Transcription; Transcription regulation.
			2(2)	2(2)				**KCTD1**	2	16.7	Q719H9	-	BTB/POZ domain-containing protein KCTD1.		Ubl conjugation.
		2(2)		2(2)				**TCOF1**	2	3.2	Q13428	Nucleoplasm; Nucleolus; Cytoplasm	Treacle protein (Treacher Collins syndrome protein).	May be involved in nucleolar-cytoplasmic transport. May play a fundamental role in early embryonic development, particularly in development of the craniofacial complex (By similarity).	Alternative splicing; Disease mutation; Nucleus; Phosphoprotein; Polymorphism; Transport.
		2(3)	3(3)					**PYCR2**	2	9.7	A6NMB5	-	Uncharacterized protein PYCR2.		
		3(6)	2(2)					**NME4**	2	36.9	O00746	Mitochondrion	Nucleoside diphosphate kinase, mitochondrial precursor (EC 2.7.4.6) (NDP kinase, mitochondrial) (NDK) (nm23-H4) (Nucleoside diphosphate kinase D) (NDPKD).	Major role in the synthesis of nucleoside triphosphates other than ATP (By similarity).	3D-structure; ATP-binding; Direct protein sequencing; Kinase; Magnesium; Metal-binding; Mitochondrion; Nucleotide metabolism; Nucleotide-binding; Phosphoprotein; Transferase; Transit peptide.
		4(4)	3(3)					**DUSP11**	2	27.6	O75319	Nucleus; Nucleolus	RNA/RNP complex-1-interacting phosphatase (EC 3.1.3.-) (Phosphatase that interacts with RNA/RNP complex 1) (Dual specificity protein phosphatase 11).	Possesses RNA 5'-triphosphatase and diphosphatase activities, but displays a poor protein-tyrosine phosphatase activity. Binds to RNA. May participate in nuclear mRNA metabolism.	Alternative splicing; Hydrolase; Nucleus; RNA-binding.
		5(6)	2(2)					**BCKDK**	2	16.7	Q96G95	Mitochondrion	BCKDK protein (Branched chain ketoacid dehydrogenase kinase, isoform CRA_a).		Kinase.
		4(5)	2(2)					**TRIM47**	2	11.8	Q96LD4	Nucleus	Tripartite motif-containing protein 47 (Gene overexpressed in astrocytoma protein) (RING finger protein 100).		Coiled coil; Cytoplasm; Metal-binding; Nucleus; Phosphoprotein; Zinc; Zinc-finger.
		2(3)						**SNX22**	2	9.3	Q96L94	-	Sorting nexin-22.	May be involved in several stages of intracellular trafficking (By similarity).	3D-structure; Protein transport; Transport.
		2(3)	2(2)					**WDR5**	2	17.7	P61964	Cytoplasm; Nucleolus	WD repeat-containing protein 5 (BMP2-induced 3-kb gene protein).	Accelerates osteoblast differentiation (By similarity).	3D-structure; Phosphoprotein; Repeat; WD repeat.
		2(2)	2(2)					**EEFSEC**	2	10.1	P57772	Cytoplasm	Selenocysteine-specific elongation factor (Elongation factor sec) (Eukaryotic elongation factor, selenocysteine-tRNA-specific).	Translation factor necessary for the incorporation of selenocysteine into proteins. It probably replaces EF-Tu for the insertion of selenocysteine directed by the UGA codon. SelB binds GTP and GDP.	Cytoplasm; GTP-binding; Nucleotide-binding; Nucleus; Protein biosynthesis.

		4(4)	5(6)					**AASS**	2	13.1	Q9UDR5	Mitochondrion	Alpha-aminoadipic semialdehyde synthase, mitochondrial precursor (LKR/SDH) [Includes: Lysine ketoglutarate reductase (EC 1.5.1.8) (LOR) (LKR); Saccharopine dehydrogenase (EC 1.5.1.9) (SDH)].	Bifunctional enzyme that catalyzes the first two steps in lysine degradation. The N-terminal and the C-terminal contain lysine-ketoglutarate reductase and saccharopine dehydrogenase activity, respectively.	Mitochondrion; Multifunctional enzyme; NAD; NADP; Oxidoreductase; Transit peptide.
		4(6)	6(6)					**GPATCH8**	2	8.7	Q9UKJ3		G patch domain-containing protein 8.		Phosphoprotein.
		2(2)	2(2)					**OSBPL9**	2	6.2	Q86YQ3	Cytoplasm	Oxysterol-binding protein-like protein 9 (Oxysterol binding protein- like 9, isoform CRA_d) (Oxysterol binding protein-like 9).		Lipid transport; Transport.
		2(2)	2(2)					**ZKSCAN4**	2	10.6	Q969J2		Zinc finger protein with KRAB and SCAN domains 4 (Zinc finger protein 307) (P373c6.1).	May be involved in transcriptional regulation.	DNA-binding; Metal-binding; Nucleus; Phosphoprotein; Repeat; Transcription; Transcription regulation; Zinc; Zinc-finger.
		5(5)					2(2)	**HELZ**	2	5.5	P42694	-	Probable helicase with zinc finger domain (EC 3.6.1.-).	May act as an helicase that plays a role in RNA metabolism in multiple tissues and organs within the developing embryo.	ATP-binding; Helicase; Hydrolase; Metal-binding; Nucleotide-binding; Nucleus; Phosphoprotein; Zinc; Zinc-finger.
		2(2)	3(3)					**TFIP11**	2	9	Q9UBB9	Nucleus; Cytoplasm; Extracellular	Tuftelin-interacting protein 11.	May play a role in the differentiation of ameloblasts and odontoblasts or in the forming of the enamel extracellular matrix. May also be involved in pre-mRNA splicing.	Alternative splicing; Biomineralization; Cytoplasm; mRNA processing; mRNA splicing; Nucleus; Phosphoprotein; Spliceosome.
			19(29)			12(18)		**TUBB2B**	2	56.9	Q9BVA1	-	Tubulin beta-2B chain.	Tubulin is the major constituent of microtubules. It binds two moles of GTP, one at an exchangeable site on the beta chain and one at a non-exchangeable site on the alpha-chain (By similarity).	Direct protein sequencing; GTP-binding; Microtubule; Nucleotide-binding.
			18(28)			11(17)		**TUBB2A**	2	48.1	Q13885		Tubulin beta-2A chain.	Tubulin is the major constituent of microtubules. It binds two moles of GTP, one at an exchangeable site on the beta chain and one at a non-exchangeable site on the alpha-chain (By similarity).	Direct protein sequencing; GTP-binding; Microtubule; Nucleotide-binding; Polymorphism.
			3(4)			2(3)		**WDR61**	2	22.6	Q9GZS3	-	WD repeat-containing protein 61 (Meiotic recombination REC14 protein homolog).		Acetylation; Repeat; WD repeat.
			2(2)					**CYLC2**	2	18.4	Q14093	Cytoplasm	Cylicin-2 (Cylicin II) (Multiple-band polypeptide II).	Possible architectural role during spermatogenesis. May be involved in spermatid differentiation.	Cytoplasm; Cytoskeleton; Developmental protein; Differentiation; Polymorphism; Repeat; Spermatogenesis; Structural protein.
			2(2)					**PCBP1**	2	16.6	Q15365	Nucleus; Cytoplasm; Nucleolus	Poly(rC)-binding protein 1 (Alpha-CP1) (hnRNP-E1) (Nucleic acid- binding protein SUB2.3).	Single-stranded nucleic acid binding protein that binds preferentially to oligo dC.	3D-structure; Cytoplasm; Direct protein sequencing; DNA-binding; Nucleus; Phosphoprotein; Repeat; Ribonucleoprotein; RNA-binding.
			2(3)				2(2)	**RIF1**	2	2.2	Q5UIP0	Nucleus	Telomere-associated protein RIF1 (Rap1-interacting factor 1 homolog).	Required for checkpoint mediated arrest of cell cycle progression in response to DNA damage during S-phase (the intra-S- phase checkpoint). This checkpoint requires activation of at least 2 parallel pathways by the ATM kinase: one involves the MRN (MRE11A-RAD50-NBS1) complex, while the second requires CHEK2. RIF1 seems to act independently of both these pathways. Seems to play no role in either the G1/S or G2/M DNA damage checkpoints.	Alternative splicing; Cell cycle; Chromosomal protein; DNA damage; Nucleus; Phosphoprotein; Polymorphism; Telomere.

			2(2)			2(3)		**KIAA1109**	2	1.8	Q4W598	-	Putative uncharacterized protein KIAA1109 (Fragment).		
						4(4)	5(6)	**RBM17**	2	31.1	Q5W011	Nucleus	RNA binding motif protein 17 (Fragment).		
						2(2)		**UBAP2L**	2	14	Q14157	Nucleus	Ubiquitin-associated protein 2-like (Protein NICE-4).		Alternative splicing; Phosphoprotein; Polymorphism.
						2(3)	2(3)	**LMNA**	2	52.4	P02545	Nucleus; Cytoplasm; Nucleolus	Lamin-A/C (70 kDa lamin) (Renal carcinoma antigen NY-REN-32).	Lamins are components of the nuclear lamina, a fibrous layer on the nucleoplasmic side of the inner nuclear membrane, which is thought to provide a framework for the nuclear envelope and may also interact with chromatin. Lamin A and C are present in equal amounts in the lamina of mammals.	3D-structure; Acetylation; Alternative splicing; Cardiomyopathy; Charcot-Marie-Tooth disease; Coiled coil; Direct protein sequencing; Disease mutation; Intermediate filament; Limb-girdle muscular dystrophy; Lipoprotein; Nucleus; Phosphoprotein; Prenylation.
						2(2)		**MTBP**	2	6.3	Q96DY7	-	Protein Mdm2-binding protein (p53-binding protein Mdm2-binding protein) (Double minute 2 protein-binding protein).		
						2(3)	3(3)	**MSH3**	2	4.3	P20585	Nucleus	DNA mismatch repair protein Msh3 (Divergent upstream protein) (DUP) (Mismatch repair protein 1) (MRP1).	Probable DNA-repair protein.	ATP-binding; DNA damage; DNA repair; DNA-binding; Nucleotide-binding; Phosphoprotein; Polymorphism.

***** number of individual peptides identified, followed by the total number of spectra collected for the protein .

Count: the count column represents the number of times the protein was identified out of all the runs.

The whole cell lysate were used for pulldown. In some cases, nuclear lysate (NL) were used instead.

TRF2-irC: sham-irradiated sample

TRF2-ir1 and TRF2-ir2 : gamma radiation-treated samples (2 Gy and 10 Gy, respectively)
